# tRNA-Dependent Import of a Transit Sequence-Less Aminoacyl-tRNA Synthetase (LeuRS2) into the Mitochondria of Arabidopsis

**DOI:** 10.3390/ijms22083808

**Published:** 2021-04-07

**Authors:** Steffen Reinbothe, Claudia Rossig, John Gray, Sachin Rustgi, Diter von Wettstein, Christiane Reinbothe, Joachim Rassow

**Affiliations:** 1 Laboratoire de Génétique Moléculaire des Plantes and Biologie Environnementale et Systémique (BEeSy), Université Grenoble-Alpes, LBFA, BP53F, 38041 Grenoble, France; claudia.rossig@gmx.de (C.R.); bt434375@myubt.de (C.R.); 2 Department of Biological Sciences, University of Toledo, 2801 West Bancroft Street, Toledo, OH 43606, USA; jgray5@utnet.utoledo.edu; 3 Department of Plant and Environmental Sciences, Clemson University Pee Dee Research and Education Center, Florence, SC 29506, USA; srustgi@clemson.edu; 4 Department of Crop and Soil Sciences, Washington State University, Pullman, WA 99164-6420, USA; diter@wsu.edu; 5 Department of Cell Biochemistry, Institute for Biochemistry and Pathobiochemistry, Ruhr-University Bochum, Universitätsstrasse 150, 44780 Bochum, Germany

**Keywords:** organelle biogenesis, mitochondria, protein transport, transit sequence-less precursors, PRAT proteins, cell death

## Abstract

Aminoacyl-tRNA synthetases (AaRS) charge tRNAs with amino acids for protein translation. In plants, cytoplasmic, mitochondrial, and chloroplast AaRS exist that are all coded for by nuclear genes and must be imported from the cytosol. In addition, only a few of the mitochondrial tRNAs needed for translation are encoded in mitochondrial DNA. Despite considerable progress made over the last few years, still little is known how the bulk of cytosolic AaRS and respective tRNAs are transported into mitochondria. Here, we report the identification of a protein complex that ties AaRS and tRNA import into the mitochondria of *Arabidopsis thaliana.* Using leucyl-tRNA synthetase 2 (LeuRS2) as a model for a mitochondrial signal peptide (MSP)-less precursor, a ≈30 kDa protein was identified that interacts with LeuRS2 during import. The protein identified is identical with a previously characterized mitochondrial protein designated HP30-2 (encoded by At3g49560) that contains a sterile alpha motif (SAM) similar to that found in RNA binding proteins. HP30-2 is part of a larger protein complex that contains with TIM22, TIM8, TIM9 and TIM10 four previously identified components of the translocase for MSP-less precursors. Lack of HP30-2 perturbed mitochondrial biogenesis and function and caused seedling lethality during greening, suggesting an essential role of HP30-2 in planta.

## 1. Introduction

Aminoacyl-tRNA synthetases (AaRS) play important roles in all eukaryotic cells [1]. Two or more AaRSs are present for each amino acid, one in the cytoplasm and the others in mitochondria and/or chloroplasts. It has been reported that many cytoplasmic and mitochondrial AaRS activities are encoded by distinct nuclear genes [2]. In other cases, single nuclear genes encode both, cytoplasmic and mitochondrial AaRS isoforms. By virtue of alternative RNA splicing or translation initiation, one form is produced that carries an NH_2_-terminal mitochondrial signal peptide (MSP) for intracellular targeting, whereas the other, shorter form lacks such extension and stays in the cytosol [3,4,5,6]. An example is provided by yeast valyl-tRNA synthetase (ValRS) [7]. Studies showed that both ValRS isoforms accomplish non-redundant roles and could not substitute for each other in vivo [7]. Chiu et al. [8] examined the evolutionary basis of converting a bacterial tRNA synthetase into a yeast cytoplasmic or mitochondrial enzyme and identified an NH_2_-terminal appendage comprising a MSP and a nonspecific tRNA-binding domain (TRBD) that act in cis and rendered the bacterial enzyme functional both in the cytoplasm and in mitochondria.

In the model (plants *Arabidopsis thaliana*), 53 genes with similarities to AaRS genes have been identified of which 24 are supposed to encode organellar enzymes, with at least 15 (probably 17) shared between mitochondria and chloroplasts and 5 shared between the cytosol and mitochondria [9,10]. Except for leucyl-tRNA synthetase 2 (LeuRS2), all organellar AaRS are predicted to contain cleavable NH_2_-terminal targeting signals [9,10]. To the best of our knowledge, LeuRS2 is the only known example of mitochondrial AaRS that lacks a predictable MSP for import. Here, we asked how import of LeuRS2 may proceed and used a combination of biochemical, cell biological, and genetic approaches to provide evidence for a pathway of tRNA-regulated protein import into mitochondria in plants.

## 2. Results

### 2.1. Identification of Proteins Interacting with LeuRS2 during Its Uptake by Isolated Arabidopsis Mitochondria

**Figure 1:**
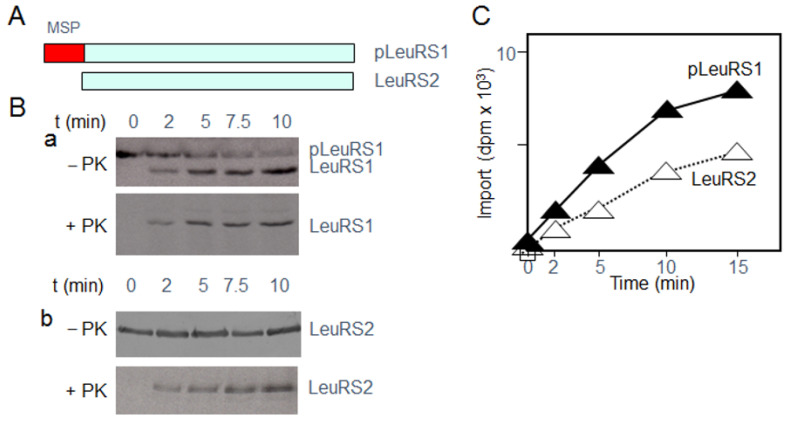
In vitro-uptake of pLeuRS1 and LeuRS2 into isolated Arabidopsis mitochondria. (**A**) Schematic representation of pLeuRS1 and LeuRS2, differing by the presence of a mitochondrial signal peptide (MSP). (**B**) In vitro-import of ^35^S-labeled pLeuRS1 (Panel a) and ^35^S-labeled LeuRS2 (Panel b) into isolated mitochondria. The autoradiogram shows ^35^S-pLeuRS1 and ^35^S-LeuRS2 in re-isolated mitochondria before (−) and after (+) proteinase K (PK) treatment. Note the processing of pLeuRS1 into the mature form lacking the mitochondrial signal peptide (MSP), whereas LeuRS2 does not undergo a processing step during import. (**C**) Time course of appearance of mature-sized LeuRS1 (black triangles) and LeuRS2 (white triangles) in proteinase K-treated mitochondria.

pLeuRS1 and LeuRS2 are distinct gene products in Arabidopsis; pLeuRS1 is encoded by At4g04350 and LeuRS2 is encoded by At1g09620. Comparison of their predicted amino acid sequences revealed common and unique features, the most significant difference being the presence of a predictable targeting signal in LeuRS1 and the absence of such signal in LeuRS2 (Figure S1; see also Figure 1A). LeuRS2 and LeuRS1 share the presence of conserved HIGH and KMSKS motives in the catalytic site and the presence of an editing domain comprising conserved Threonine-rich and GTG motives needed for high-fidelity aminoacylation of tRNA (Figure S1). A degenerate DWLISR signature motif is present in both pLeuRS1 and LeuRS2 (Figure S1). Unlike LeuRS1 and other LeuRS, LeuRS2 lacks canonical Zn and leucine-specific domains (Figure S1).

cDNA clones were generated and used for coupled in vitro-transcription/translation to produce radiolabeled pLeuRS1 and LeuRS2 proteins for mitochondrial import assays. Figure 1B,C show that ^35^S-methionine-labeled pLeuRS1 was readily taken up by isolated mitochondria and processed to mature size, whereas LeuRS2 was imported but not processed. This result is consistent with the presence of a cleavable MSP in pLeuRS1 and the lack of such sequence in LeuRS2 (cf. Figure S1). Resistance towards proteinase K (Figure 1B) confirmed the location of LeuRS1 and LeuRS2 in mitochondria. As shown previously, mitochondrial precursors produce protease-resistant forms when imported into mitochondria, whereas unimported proteins do not and are rapidly degraded by added protease [11,12]. 

**Figure 2:**
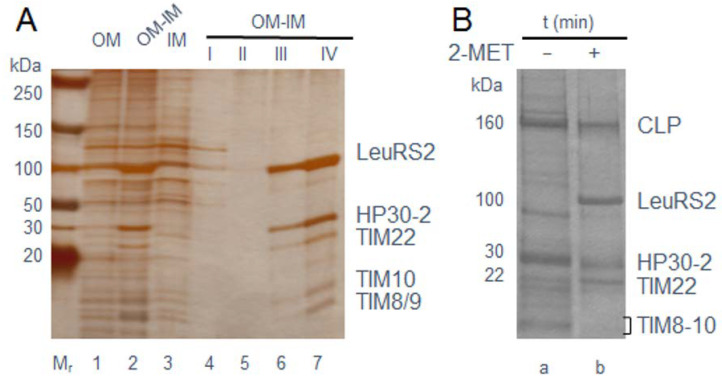
Identification of proteins interacting with LeuRS2 during its import into mitochondria. (**A**) Detection of LeuRS2-(His)_6_ in translocation intermediates produced at 8 °C during an in vitro-uptake reaction with isolated mitochondria. Lanes 1–3, patterns of proteins found in outer membranes (OM), inner membranes (IM) and OM–IM fractions obtained in the presence of imported LeuRS2-(His)_6_ after sucrose density gradient fractionation of ruptured mitochondria. Fractions I, II, III and IV (Lanes 4–7) represent the different washing and elution fractions of the detergent-solubilized OM–IM fraction containing LeuRS2-(His)_6_ during affinity purification on Ni-NTA agarose. Among the co-purifying proteins are HP30–2, TIM22 as well as TIM8/9 and TIM10. Positions of molecular mass standards (M_r_) are indicated to the left. (**B**) Co-isolation of DTNB-activated LeuRS2-(His)_6_ with HP30-2, TIM22, TIM8/9 and TIM10 as well as other, unidentified bands in OM–IM fractions (− 2-MET, Lane a) and cleavage of the established higher molecular mass crosslink product (CLP) of ≈160 kDa into LeuRS2-(His)_6_, HP30-2 and TIM22 by 2-mercaptoethanol (+ 2-MET, Lane b).

We next identified proteins interacting with LeuRS2 in mitochondria during its import. Bacterially expressed LeuRS2 containing a hexa-histidine-tag for purification (LeuRS2-[His]_6_) was added to Arabidopsis mitochondria. As shown previously, import of proteins into the mitochondrial matrix (the location expected for LeuRS2) requires ATP and a membrane potential ∆Ψ across the inner mitochondrial membrane (see [13], for review). Translocation slows when carried out at reduced temperature [14], allowing the isolation of translocation intermediates. Figure 2A shows that incubation of LeuRS2 with isolated mitochondria at 8 °C gave rise to an intermediate density fraction (referred to as outer membranes (OM)–inner membranes (IM) (OM–IM fraction) obtained after centrifugation of ruptured mitochondria on sucrose gradients [15]. This OM–IM fraction comprised both outer and inner mitochondrial membrane proteins presumably held together in contact sites (Figure 2A, Lanes 1–3). After purifying LeuRS2-(His)_6_ from detergent-solubilized OM–IM fractions, 4 protein bands were identified by SDS-PAGE and protein sequencing (Figure 2A, Lanes 4–7; see also SI materials). These bands comprised a protein previously referred to as ‘hypothetical protein of 30 kDa’ (designated HP30-2) dually localized in chloroplasts and mitochondria [16,17], the 22 kDa translocon protein of the inner mitochondrial membrane TIM22 (encoded by At3g10110 or At1g18320), as well as TIM10 (encoded by At2g29530), TIM9 (encoded by At3g46560) and TIM8 (encoded by At5g50810), whose orthologues in yeast all operating in the uptake of MSP-less mitochondrial precursors such as carrier and β-barrel proteins [17,18]. Crosslinking with Ellman’s reagent [5,5’-dithiobis-(2-nitrobenzoic acid), DTNB] [19] proved tight interactions between LeuRS2-(His)_6_ and HP30-2 and additionally demonstrated their co-purification with TIM22, TIM8, TIM9 and TIM10 (Figure 2B, − 2-MET [2-mercaptoethanol]). After purification from SDS-dissolved OM–IM fractions, the main, ≈160 kDa crosslink product was cleaved by 2-mercaptoethanol (Figure 2B, + 2-MET) into LeuRS2-(His)_6_, HP30-2 and TIM22 (Figure 2B, + 2-MET), indicating the presence of mixed disulfide bonds of LeuRS2 with HP30-2 and TIM22 that could be explained by formation of a membrane channel in which LeuRS2 is engaged. Co-immunoprecipitation experiments confirmed the presence of HP30-2 and TIM22 in the established crosslink product of LeuRS2-(His)_6_ (Figure S2). When translocation intermediates were prepared for pLeuRS1-(His)_6_, a different protein pattern was obtained, comprising, amongst other proteins, TIM23 and TIM17-2 (Figure S3) and thus indicating that pLeuRS1-(His)_6_ used a different import pathway than LeuRS2-(His)_6_. 

### 2.2. Import of LeuRS2 into Mutant Mitochondria Deprived of HP30-2

**Figure 3:**
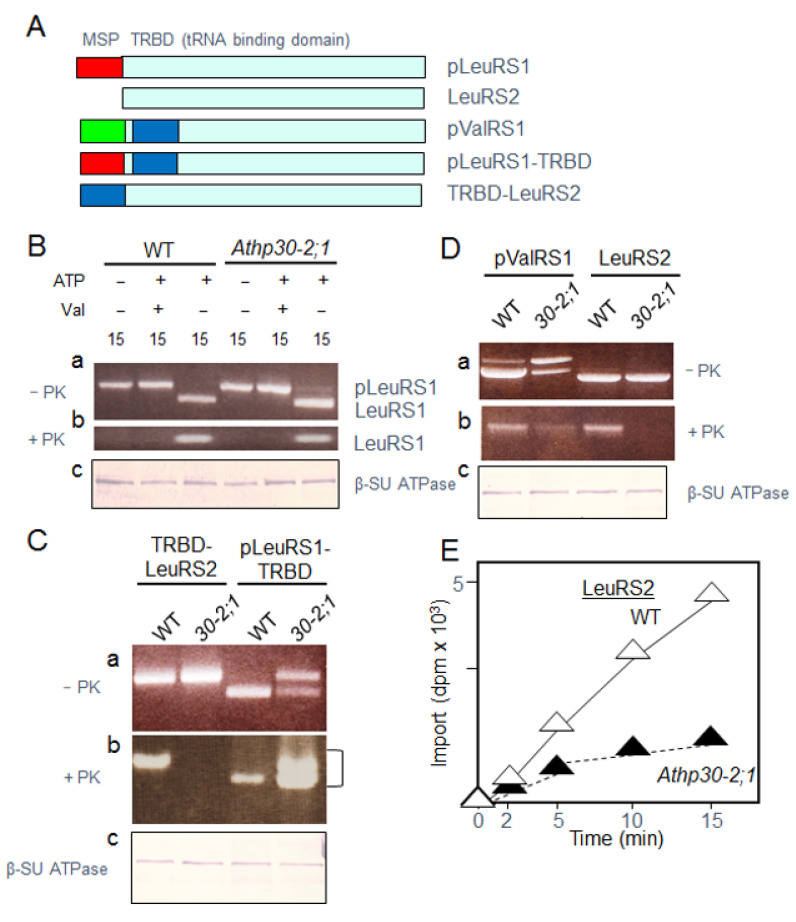
Mitochondrial import of pLeuRS1, LeuRS2, pValRS1 and engineered versions of pLeuRS1 and LeuRS2 containing the tRNA binding domain (TRBD, blue box) present in pValRS1. (**A**) Structure of the different AaRSs (not drawn to scale). MSP defines the mitochondrial signal peptide of pLeuRS1 (red box) and pValRS1 (green box). (**B**) Import of pLeuRS1 into mitochondria of wild-type (WT) and *Athp30-2;1* plants. The assays were conducted at 23 °C and contained ATP and valinomycine (Val), as indicated. Parallel assays were treated with (+ Prot. K) or without proteinase K (-Prot. K). Panels (**a,b**) show precursor and mature protein levels before (−) and after (+) proteinase K (Prot. K) treatment. Panel (**c**) depicts the result of a Western blot that was probed with an antibody against the β-subunit of the mitochondrial CF_0_/CF_1_-ATPase (β-SU ATPase). (**C**) as (**B**), but showing the results of an import reaction carried out with TRBD-LeuRS2 and pLeuRS1-TRBD for mitochondria of wild-type (WT) and *Athp30-2;1* plants. Incubations contained ATP but lacked valinomycin. (**D**) as (**C**), but showing the results of an import reaction conducted with pValRS1 and LeuRS2 for mitochondria of wild-type (WT) and *Athp30-2;1* plants. (**E**) Time course of LeuRS2 import into mitochondria of wild-type (WT) and *Athp30-2;1* plants.

Mutants were identified for At5g24650 (HP30-2) and At3g49560 (HP30) (Figures S4 and S5) and used for mitochondrial in vitro-uptake assays with LeuRS2. For comparison, we used pLeuRS1 and pValRS1, two AaRS precursors synthesized with cleavable NH_2_-terminal MSPs [9,10]. Data presented in Figure S4D (Panel b) demonstrated a lack of import of LeuRS2 into mitochondria of *Athp30-2;1* plants. By contrast, no such import defect was observed for pLeuRS1 (Figure S4D), excluding the possibility of an indirect effect of the *hp30-2* mutation on the ∆Ψ needed for the translocation of proteins into the matrix. In tRNA uptake assays, no differences were observed for *Athp30-2;1, Athp30-2;2* versus wild-type mitochondria (Figures S6–S8). This result excluded a role of HP30-2 as tRNA transporter. Interestingly, import of pValRS1 occurred with similar efficiencies for mitochondria from wild-type and *Athp30-2;1* plants, except for the appearance of a partially processed form migrating between the precursor and mature forms (Figure S4D, Panel b, arrowhead). This processing intermediate was no longer observed in genetically complemented *Athp30-2;1* plants (Figure S4D, Panel b). Its appearance was reminiscent of that of yeast pValRS1 that contains a bipartite NH_2_-terminal extension consisting of an NH_2_-terminal MSP and close-by non-specific tRNA binding domain (TRBD) (Figure 3A) regulating its intracellular targeting and activity [8]. A database search of yeast pValRS1 (AAA35207) highlighted the presence of a similar, yet truncated TRBD in pValRS1 (G41-Q135, coded for by At1g14610) in *Arabidopsis thaliana* (Figure S9).

Constructions were made to place the TRBD of yeast pValRS1 into pLeuRS1 of Arabidopsis to produce pLeuRS1-TRBD (Figure 3A). On the other hand, we engineered a fusion protein consisting of the TRBD in front of LeuRS2 to produce TRBD-LeuRS2 (Figure 3A). Radiolabeled precursors were synthesized in vitro from the generated cDNAs and used in mitochondrial protein import assays. These were terminated by re-isolating intact mitochondria and treating them with proteinase K. Data in Figure 3B demonstrated pLeuRS1 import into mitochondria of both wild-type and *Athp30-2;1* plants; obviously, this import was HP30-2-independent. For LeuRS2, uptake was undetectable for *Athp30-2;1* mitochondria, as compared to wild-type mitochondria (Figure 3D,E). Addition of the TRBD in front of LeuRS2 did not restore import of TRBD-LeuRS2 into *Athp30-2;1* mitochondria (Figure 3C). For pLeuRS1-TRBD, reductions of import were observed for *Athp30-2;1* plants, as deduced from the lower amount of mature-sized LeuRS1-TRBD in proteinase K-treated mitochondria (Figure 3C, Panels a and b). Because unprocessed pLeuRS1-TRBD was detectable in re-isolated, proteinase K-treated *Athp30-2;1* mitochondria (smear in Figure 3C, Panel b), we concluded that a portion of added pLeuRS1-TRBD had traversed the outer mitochondrial membrane but was not imported further. Similar to pLeuRS1-TRBD, uptake of yeast pValRS1 was reduced for mitochondria from *Athp30-2;1* plants, as compared to wild-type plants, although no intermediate-sized form appeared (Figure 3D). This result suggested that yeast pValRS1 may not undergo double-processing in the assays with plant mitochondria. On the other hand, some crossover of import pathways could occur, with HP30-2 binding MSP-less precursors, such as LeuRS2, as well as MSP-containing precursors possessing TRBDs such as plant and yeast pValRS1.

**Figure 4:**
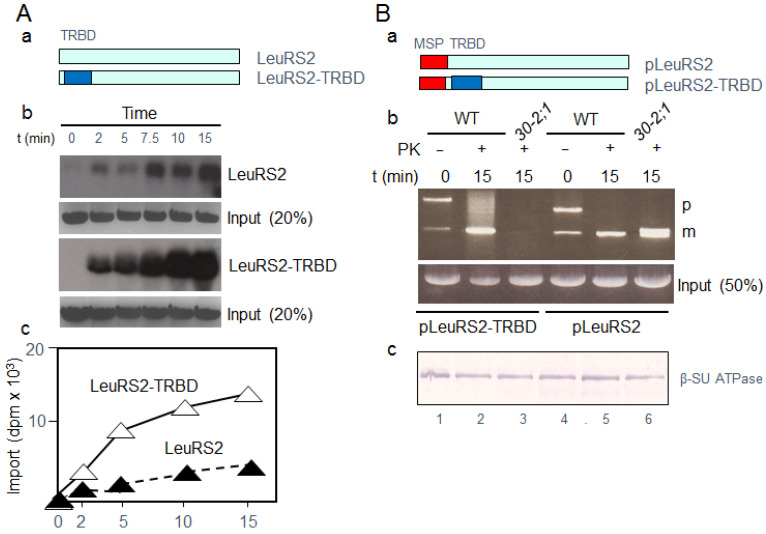
Mitochondrial import of LeuRS2 and engineered versions of LeuRS2 containing the mitochondrial presequence (MSP) of pLeuRS1 (pLeuRS2) and the tRNA binding domain (TRBD) of yeast pValRS1. (**A**) Structure of LeuRS2 and LeuRS2-TRBD (**a**) and import into mitochondria of wild-type plants (**b**,**c**). In Panel b, respectively, 20% import standards are included for LeuRS2 and LeuRS2-TRBD. (**B**) Structure of engineered pLeuRS2 containing or lacking the TRBD (**a**) and import into mitochondria of wild-type (WT) and *Athp30-2;1* plants (**b**). In Panel b, a respective, 50% import standard is shown for each lane. PK, proteinase K. Panel c shows a replicate gel blot traced with an antibody against the β-subunit of the mitochondrial CF_0_/CF_1_-ATPase (β-SU ATPase).

To explore this hypothesis, we engineered LeuRS2-TRBD fusion proteins containing or lacking pLeuRS1′s MSP, giving rise to pLeuRS2-TRBD and LeuRS2-TRBD (Figure 4B). In pilot experiments that were terminated by the addition of proteinase K, radiolabeled LeuRS2 and LeuRS2-TRBD protein lacking pLeuRS1′s MSP (Figure 4A) were added to wild-type and *Athp30-2;1* mitochondria in standard uptake assays. When time courses over import were compared, significantly more ^35^S-LeuRS2-TRBD than ^35^S-LeuRS2 was imported into wild-type mitochondria (Figure 4A). When import of LeuRS2 fusion proteins containing pLeuRS1′s MSP was analyzed, remarkable differences became apparent for ^35^S-pLeuRS2-TRBD and ^35^S-pLeuRS2 (Figure 4B). Whereas ^35^S-pLeuRS2 was imported into mitochondria from *Athp30-2;1* plants, ^35^S-pLeuRS2-TRBD was not. These results indicated that the NH_2_-terminal MSP and TRBD differentially regulated mitochondrial protein import.

### 2.3. Evidence for a tRNA Requirement of LeuRS2 Import

**Figure 5:**
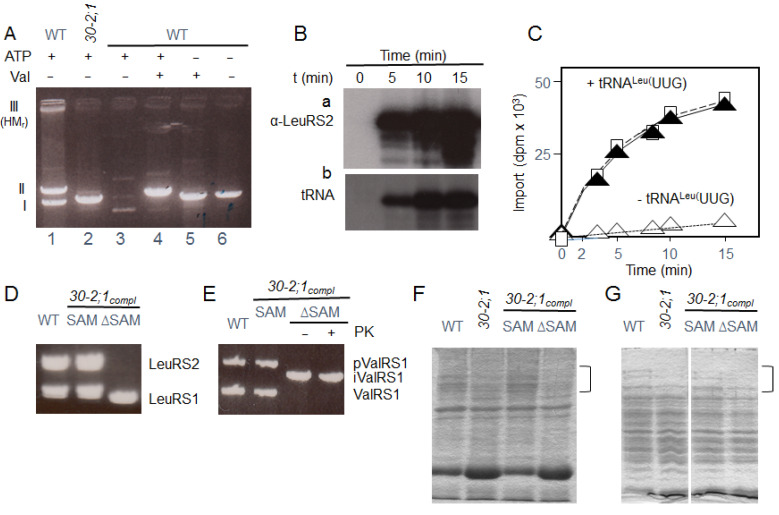
LeuRS2-tRNA co-import and effect of the sterile alpha motif (SAM) domain of HP30-2. (**A**) Agarose gel electrophoresis to detect LeuRS2-(His)_6_-bound tRNA in mitochondrial translocation intermediates in the wild-type (WT) and *Athp30-2;1* (SALK_136524) T-DNA insertion mutant at the indicated combinations of ATP and valinomycin. Incubations were carried out at 8 °C. Note the lack of any of these bands in wild-type mitochondria that had been pretreated with proteinase K (Lane 3). (**B**) Time course of LeuRS-(His)_6_ and tRNA uptake into the matrix of wild-type mitochondria. Incubations were carried out at 23 °C and the amounts of LeuRS2-(His)_6_ and tRNA determined by successive steps of western and northern blotting, respectively, carried out on the same nitrocellulose membrane. (**C**) as B, but showing kinetic LeuRS2 uptake data for tRNA^Leu^(UUG)-containing assays (black triangles) and tRNA^Leu^(UUG)-free assays (white triangles). For comparison, tRNA^Leu^(UUG) uptake data were included (white squares). D and E, as B, but showing the amount of LeuRS1-tRNA and LeuRS2-tRNA (**D**) versus ValRS1-tRNA (**E**) imported into mitochondria of WT plants and complemented *hp30-2;1* plants (*hp30-2;1_compl_)* expressing either the full-length HP30-2 cDNA (SAM) or cDNA lacking the coding sequence of the SAM domain (∆SAM). Note the accumulation of intermediate-sized ValRS1 (iValRS1) in mitochondria of *hp30-2;1* plants expressing the SAM-less variant of HP30-2. PK, proteinase K. F and G, patterns of total mitochondrial proteins (**F**) and soluble matrix proteins (**G**) in WT plants and WT plants expressing either the full-length HP30-2 (SAM) or HP30-2∆SAM cDNAs.

LeuRS2-(His)_6_ was imported at 8 °C into isolated Arabidopsis wild-type mitochondria in assays containing ATP. To monitor the binding of tRNA to LeuRS2, the assays additionally contained mixtures of fluorescence-labeled cytosolic tRNAs. Translocation intermediates were isolated as described and separated by non-denaturing PAGE. After electrophoresis, three main tRNA-containing bands were visible, one higher molecular mass band migrating near the depot region of the gel (referred to as Band III) and most likely representing LeuRS2-(His)_6_ assembled with other proteins including HP30-2 and TIM22 as well as tRNA^Leu^ into larger complexes (Figure S10A,B, Lane 1), and two lower molecular mass bands (referred to as Band I and Band II) (Figure 5A, Lane 1). Because only Band I was seen in import reactions carried out with mitochondria from *Athp30-2;1* plants, this band is likely due to LeuRS2-(His)_6_ bound to, but not imported into, mitochondria (Figure 5A, Lane 2). In line with this view, the appearance of Band I was undetectable for wild-type mitochondria that had been pretreated with proteinase K (Figure 5A, Lane 3). By contrast, Band II may represent an early translocation intermediate formed between LeuRS2-(His)_6_ and components of the outer mitochondrial membrane (Figure S10A,B Lanes 2 and 3). It is noteworthy that this band was not observed in the initial experiments and may be due to the presence of tRNA in the uptake assays, presumably hindering translocation and leading to a transient translocation arrest. The appearance of this early translocation intermediate was strongly favored by valinomycin that is known to dissipate the membrane potential ∆Ψ needed for translocation of cytoplasmic proteins across the inner mitochondrial membrane (Figure 5A, Lane 4). Neither Band II nor Band III were detectable in incubations mixtures lacking ATP, regardless of whether or not these contained valinomycin, a result that underscored the ATP requirement of import (Figure 5A, Lanes 5 and 6). When translocation of LeuRS2-(His)_6_ was allowed to proceed at 23 °C in the presence of ATP, no translocation intermediates were detected and LeuRS2 was imported into the matrix (Figure 5B, Panel a). Fluorescence analyses confirmed binding of tRNA to the imported mitochondrial LeuRS2 (Figure 5B, Panel b). Quantitation of import data showed that the 6 tRNA^Leu^ isoacceptors, that had been isolated from the tRNA mixture, differentially stimulated uptake of LeuRS2, as compared to tRNA-free assays (Table S1). Respective controls with yeast tRNA^Ser^ and bacterial tRNA^Tyr^ isoacceptors failed to stimulate mitochondrial LeuRS2 import (Table S2). This result is consistent with the stringent substrate specificity and high-fidelity editing capability of LeuRS2, distinguishing both the A73 discriminator and base composition of the long variable arm in these three tRNAs. Because all tested tRNA^Leu^, tRNA^Ser^ and tRNA^Tyr^ isoacceptors were taken up with similar rates by isolated wild-type, *hp30-2;1* and *hp30-2;2* mitochondria in assays lacking the LeuRS2 carrier protein (Table S3), we concluded that their import per se is HP30-2-independent. Additional in vitro-mutagenesis studies using LeuRS2 derivatives that had been deprived of their HIGH and KMSKS motives in the catalytic site and Threonine-rich and GTG signature motives in the editing domain confirmed the specificity of the tRNA^Leu^::LeuRS2 interaction during their mitochondrial co-import (Table S4).

HP30-2 contains a sterile alpha motif (SAM) motif that is related to the one found in RNA binding proteins such as Smaug from *Drosophila melanogaster* and Vts1 from *Saccharomyces cerevisiae* [20,21]. We hypothesized that this SAM could provide a sort of tRNA relay that unloads and reloads tRNA during LeuRS2′s transport across the inner mitochondrial membrane. Transgenic *Athp30-2;1* plants were generated expressing SAM-free HP30-2 (Figure S11). When mitochondrial protein import assays were run with LeuRS2, pLeuRS1 and pValRS1, *Athp30-2;1* plants expressing the HP30-2∆SAM cDNA and thus contained SAM-free HP30-2 (*Athp30-2;1::*HP30-2∆SAM) were unable to import LeuRS2 but still imported pLeuRS1 (Figure 5E,F). For pValRS1, import and processing at first glance seemed to occur with similar rates for mitochondria of *Athp30-2;1::*HP30-2SAM and *Athp30-2;1::*HP30-2∆SAM plants versus wild-type plants. However, only the previously identified processing intermediate (cf. Figure S4D, Panel b) accumulated in mitochondria of *Athp30-2;1::*HP30-2∆SAM plants (Figure 5E,F). Coomassie staining of total mitochondrial and soluble matrix proteins revealed additional changes in the protein patterns in *Athp30-2;1::*HP30-2∆SAM versus *Athp30-2;1::*HP30-2SAM plants (Figure 5E,F). In summary, the results suggested a function of HP30-2′s SAM for import of LeuRS2 and TRBD-containing precursors, such as ValRS1, into mitochondria in planta.

## 3. Discussion

The mitochondrial genome of Arabidopsis encodes 22 tRNAs: however, these are insufficient to decode the entire set of codons found in the mitochondrial genome. In fact, tRNAs for 6 amino acids are missing in Arabidopsis mitochondria, including those for leucine, necessitating some tRNA import to occur in planta. Broadly, two mechanisms of mitochondrial tRNA import have been discussed [22,23]. In protozoans, membrane-bound tRNA transporters are supposed to operate [24]. In yeast, tRNAs are thought to be co-imported with proteins along the general protein import pathway [25]. In humans, both mechanisms are operative [26,27]. For plants, the situation is less well characterized [28,29,30]. Murcha et al. [31] put forth that HP30 and/or HP30-2 as PRAT protein family members containing conserved SAM RNA binding domains could act as mitochondrial tRNA transporters. Mutants lacking HP30 and HP30-2 exhibited growth reductions when grown in a 16h-photoperiod that were attributed to mitochondrial tRNA import defects [31]. However, tRNA uptake assays used to support this conclusion all were carried out in the presence of a carrier protein, Su9-DHFR, consisting of the presequence of Subunit 9 of mitochondrial ATP synthase from *Neurospora crassa* linked to the dihydrofolate reductase of mouse [31]. To the best of our knowledge, no data were presented for Su9-DHFR-free assays that would have allowed proving the function of HP30-2 and HP30 as tRNA transporters [31]. On the other hand, previous work failed to confirm the localization of HP30 in isolated Arabidopsis mitochondria [32]. Last but not least, fundamental differences were observed with regard to the sub-localization, topology and function of HP30-2 in mitochondria [31,32] such that the study of Murcha et al. [31] need to be considered with caution.

We observed that HP30-2 is part of a unique protein translocase in plant mitochondria that contains with TIM22, TIM10 and TIM8 three TIM four components previously identified as being involved in the import of MSP-less hydrophobic carrier proteins and β-barrel proteins into mitochondria [13]. The HP30-2-TIM22 complex ties tRNA to LeuRS2 import and thus appears to be part of a unique co-import mechanism. In line with this view are experiments showing that mutants lacking HP30-2 were largely incapable of co-importing LeuRS2 and mt-tRNA^Leu^ (UUR), two of the six tRNA^Leu^ isoacceptors. Results obtained for LeuRS2-free assays revealed no import defects per se for any of the tested plant, yeast and bacterial tRNAs and *hp30-2;1* and *hp30-2:2* mitochondria (Figures S6–S8) such that a role of HP30-2 as tRNA transporter seems rather unlikely.

Interestingly, previous in vivo studies identified mutations in a tRNA that prevented both its mitochondrial import and aminoacylation (28–30). On the other hand, in vitro-transcribed tRNA was shown to be taken up by isolated mitochondria from potato in an ATP-dependent and membrane potential-dependent manner (28–30). This in vitro-uptake of tRNAs could be inhibited by monospecific antibodies against the voltage-dependent anion channel (VDAC) (36–33) and was also sensitive to antibodies against TOM20 and TOM40 [30], two components of the TOM complex operating in mitochondrial protein import [13]. It was proposed that TOM20 and TOM40 could act as primary tRNA receptors, whereas VDAC could establish actual tRNA import pore [30]. Such a scenario would explain why mitochondria from *Athp30-2;1* and *Athp30-2;*2 plants imported all tested tRNAs with similar rates as those in wild-type plants (Figures S6–S8). Whereas VDAC is an outer mitochondrial membrane protein [33], HP30-2 was found to be an inner mitochondrial membrane protein [32]. UV light-induced crosslinking of each of the two tRNA^Leu^UUR isoacceptors to LeuRS2 blocked mitochondrial uptake of both, LeuRS2 and tRNA^Leu^UUR, when carried out prior to the import reaction, while crosslinking induced during the import reaction labeled HP30-2 and TIM as main products (Figure S12). On the basis of these findings, it is tempting to conclude that LeuRS2 needs to unfold and to release its bound tRNA shortly before or during translocation. We assume that HP30-2, by virtue of its SAM domain, transiently binds the tRNA and thereby provides a relay for reloading it onto LeuRS2 once respective domains of the polypeptide chain become available at the exit site of the import channel (Figure S13, Route a). Whether HP30-2 represents the actual translocation pore or a type of TIM22 complex organizer is not yet clear and needs to be examined in future work. On the other hand, we cannot entirely exclude that tRNA^Leu^ that is stripped from LeuRS2 is imported across the outer mitochondrial membrane via VDAC and then binds HP30-2 (Figure S13, Route b). Both scenarios are consistent with the results presented here. Support for an essential role of the SAM domain in the HP30-2-dependent co-import pathway of LeuRS2 and tRNA^Leu^ comes from studies using transgenic *Athp30-2;1* plants expressing HP30-2 deprived of its SAM domain that were largely incapable of importing LeuRS2 (Figure 5D,E). Vice versa, LeuRS2 mutant proteins lacking distinct parts of the catalytic site and editing domain were largely impaired in the tRNA^Leu^-dependent import (Table S4) while retaining their gross overall 3D-structures, as revealed by structural modelling (Figures S14–S21). *Athp30-2;1::*HP30-2∆SAM plants additionally accumulated partially processed pValRS1, indicating that the HP30-2-dependent pathway may also be involved in the uptake of MSP-containing precursors with TRBDs. Evidence provided elsewhere demonstrates an even larger, general role of HP30-2 in the transport of MSP-less precursor proteins [32].

The need for coupling LeuRS2 and tRNA^Leu^ import could have different reasons. For example, the most frequent and extensively studied mitochondrial (mt) tRNA mutation in humans is m.3243A→G, one of the 32 disease-associated mutations within the *MTTL1* gene coding for mt-tRNA^Leu^ (UUR) (https://www.mitomap.org/foswiki/bin/view/MITOMAP/MutationsRNA; accessed on: 23 March 2021) (see also [34,35]) that leads to a wide range of currently untreatable disorders. Interestingly, human LeuRS or short peptides derived thereof (β30_31 and β32_33) were able to rescue the disease-causing mitochondrial tRNA defect caused by m.3243A→G as well as m.8344A→G [36]. In vitro evidence suggests that these peptides bind with high affinity wild-type and mutant mt-tRNA^Leu^ (UUR) and thereby stabilize mutated mt-tRNA^Leu^(UUR) [36], presumably to dampen the penetrance of the homoplasmic disease. It is intriguing to note that in our experiments the tRNA^Leu^UUR isoacceptors were more stimulatory for the import of LeuRS2 into mitochondria than the tRNA^Leu^CUR isoacceptors. This suggests some thus far unrecognized role of the anticodon loop in tRNA recognition by LeuRS2 in Arabidopsis. Previous work carried out for other LeuRS had suggested the long variable arm and discriminator A73 to provide major identity elements of tRNA^Leu^ [37]. On the other hand, it is tempting to conclude that the need for tRNA^Leu^UUR in plant mitochondria might be a reason why the import of this tRNA is tied to that of LeuRS2. These and other studies highlighting non-canonical roles of tRNAs in protein and phospholipid modifications could explain why many pathological mutations in mitochondrial tRNAs and associated processes cause disorders and disease [38,39]. Other data place tRNA into the context of apoptotic cell death regulation (see Refs. [40,41], for review). It has been reported, for example, that tRNA can bind to, and thereby prevent, the release of cytochrome c from mammalian mitochondria into the cytosol, which normally triggers caspase activation and apoptotic cell death [40,41]. It is attractive to hypothesize that the conditional cell death phenotype leading to seedling lethality in *Athp30-2;1* plants during greening could be due to such mechanism. Work is needed to dissect the pathway of cell death regulation in *Athp30-2;1* plants.

## 4. Materials and Methods

### 4.1. Plant Materials and Growth Conditions

Genes, knock-out mutant and other plant materials used in this study were the followings: ecotype Columbia (Col-0; referred to as wild-type); *Athp30;2* (SALK_112126), *Athp30;3* (SALK_046194), *Athp30;4* (SALK_031707)*; Athp30-2;0* (SALK_149871), *Athp30-2;1* (SALK_136524), *Athp30-2;2* (SALK_136525) [42]; *Athp30;2* (SALK_112126)::*Athp30-2;1* (SALK_136524) double mutants. *RNAi#2* lacking HP30 and HP30-2 simultaneously has been described elsewhere [32]. Plants were grown at 23 °C under long-day conditions (16 h at 100 μE m^−2^ s^−1^ light, 8 h dark), except for *Athp30-2* knock-out mutants as well as *Athp30;2* (SALK_112126)::*Athp30-2;1* (SALK_136524) double mutants and *Athp30::Athp30-2* RNAi plants [32] that were cultivated under continuous white light illumination. For illumination experiments, surface-sterilized seeds were germinated on sucrose-free, half-strength Murashige–Skoog (MS)-agar medium (Merck KGaA, Darmstadt, Germany) and grown in darkness for 4.5 d, before being exposed to white light of ≈210 µE m^−2^ s^−1^ [32].

### 4.2. tRNA Isolation and Uptake Assays 

tRNA was isolated, ^32^P- or fluorescence-labeled and used for standard uptake assays, as described [32].

### 4.3. Organelle Isolation, Protein Synthesis and Protein Import Assays 

Intact mitochondria were isolated as described [32] and dissolved in a buffer containing 20 mM HEPES-KOH, 0.6 M sorbitol (pH 7.2) at a protein concentration of 10 mg/mL. Carboxy-terminally or NH_2_-terminally, hexa-histidine (His)_6_-tagged, ^35^S-methionine-labeled pLeuRS1, LeuRS2 and pValRS1 and respective derivatives were produced in *Escherichia coli* strain SG13009 (Qiagen, Hilden, Germany), purified by Ni-NTA agarose chromatography to ≈85–90% purity and added to isolated mitochondria for standard protein import assays [32]. After incubation, intact mitochondria were re-isolated and treated with proteinase K [32].

### 4.4. Isolation of Translocation Intermediates of LeuRS2 

Translocation intermediates of LeuRS2-(His)_6_ were prepared as described by lowering the reaction temperature of the import assays from 23 °C to 8 °C [14]. Isolation of translocation intermediates was achieved by rupturing mitochondria and loading crude membrane fractions on 20–38% (*w*/*v*) sucrose gradients [32]. LeuRS2-(His)_6_-containing complexes were isolated from maltoside-solubilized OM–IM junction complexes by affinity chromatography on Ni-NTA agarose (Cube Biotech GmbH, Monheim, Germany), followed by Coomassie or silver staining and protein sequencing (see below). Crosslinking of DTNB-activated LeuRS2-(His)_6_ to mitochondrial proteins was achieved as described [19], followed by affinity purification of crosslink products from SDS-dissociated OM–IM fractions. To cleave the established mixed disulfide bonds between LeuRS2-(His)_6_ and near-by mitochondrial proteins (Merck KGaA, Darmstadt, Germany), the samples were treated with 2-mercaptoethanol [32].

### 4.5. Protein Analyses

Total mitochondria or inner mitochondrial membranes were dissolved in doubly-concentrated SDS sample buffer and separated on 12% SDS-PAGE gels as described [32] or subjected to agarose gel electrophoresis [43]. Western blotting was carried out according to Towbin et al. [44] using specific antibodies and alkaline phosphatase-based detection with BCIP (5-bromo-4-chloro-3-indolyl phosphate) and NBT (nitro blue tetrazolium) or enhanced chemiluminescence (ECL Western Blotting Analysis system, Amersham, Merck KGaA, Darmstadt, Germany). For antibody production, either bacterially expressed proteins that had been produced in *Escherichia coli* or proteins that had been isolated from import intermediate-associated proteins of pLeuRS1-(His)_6_ or LeuRS2-(His)_6_ were used. The different antibodies then were tested with either total leaf protein or mitochondrial protein extracts from 14d-old plants; respective preimmune sera (PIS) were included as control. For reference, antibodies against TIM8, TIM9 and the beta subunit of the mitochondrial ATPase were purchased from Agrisera AB, Vännäs, Sweden. Production of antibodies against HP30-2 has been described elsewhere [32]. Protein was stained with Coomassie Brilliant Blue G25 (Merck KGaA, Darmstadt, Germany) and subjected to automated or manual sequencing [45].

### 4.6. Cell Death Measurements

DNA breakage indicative of apoptotic cell death was measured by agarose gel electrophoresis and ethidium bromide staining [32].

### 4.7. Structural Modelling

Multiple sequence alignments and 3D-structural modeling were conducted using standard algorithms including the Clustal Omega (https://www.ebi.ac.uk/Tools/msa/clustalo/, accessed on 5 February 2021), SWISS-MODEL (https://swissmodel.expasy.org/interactive, accessed on 5 February 2021) and I-TASSER (https://zhanglab.ccmb.med.umich.edu/I-TASSER/, accessed on 5 February 2021) online protein alignment, structure and function prediction tools.

## 5. Conclusions

Mitochondria play important roles in mammalian cells. Dysfunction of mitochondrial biogenesis and activity causes a variety of diseases and is involved in many metabolic disorders For example, lack of the mammalian mRNA binding protein regulator Smaug1/Samd4 impedes synapse plasticity and muscle function. Mitochondrial tRNA (mt-tRNA) genes provide additional ‘hot spots’ for pathological mutations provoking various disease states. Often, these mutations prevent tRNA aminoacylation but recent evidences suggest additional, non-canonical roles of tRNAs in apoptotic cell death regulation. Here, we identified a protein that mediates the tRNA-dependent mitochondrial import of a cytosolic aminoacyl-tRNA synthetase in the model plant Arabidopsis plants and show that its absence provokes growth defects and cell death.

## Data Availability

Data is contained within the article and respective Supplementary Materials.

## Supplementary Materials

Supplementary materials can be found at https://www.mdpi.com/article/10.3390/ijms22083808/s1.

## Abbreviations

AaRS	aminoacyl tRNA synthetase
MSP	mitochondrial signal peptide
PRAT	preprotein and amino acid transporter
SAM	sterile alpha motif
TIM	translocase of the inner mitochondrial membrane
TRBD	tRNA binding domain
