# Supplementary material for: tRNA-Dependent Import of a Transit Sequence-Less Aminoacyl-tRNA Synthetase (LeuRS2) into the Mitochondria of Arabidopsis
Source: Int J Mol Sci. 2021 Apr 7;22(8):3808. doi: 10.3390/ijms22083808 (PMC8067559; doi:10.3390/ijms22083808)
Supplement: Supplementary file 1 [file ijms-22-03808-s001.pdf]

SI Results

Identification of Knock-Out Mutants and RNAi Lines for HP30-2. HP30-2 is a member of the preprotein and amino acid transporter (PRAT) family that comprises 16 members in Arabidopsis (1,2). Two closely related genes are present in the Arabidopsis genome encoding HP30-like proteins, *HP30* and *HP30-2*. HP30-2 is encoded by At5g24650, whereas HP30 is encoded by At3g49560 (1,2). HP30-2 and HP30 share an overall 79% amino acid sequence identity and both contain conserved sterile alpha motives (SAMs) present in RNA binding proteins such as Smaug from *Drosophila melanogaster* and Vts1 from *Saccharomyces cerevisiae* (3,4). Murcha et al. (5) proposed a function of HP30-2 and HP30 as mitochondrial tRNA transporters.

A genetic approach was undertaken to define the roles of HP30-2 and HP30 in planta. Knock-out mutants were identified for At5g24650 (HP30-2) and At3g49560 (HP30) and characterized further (Fig. S4A). In addition, previously characterized knock-out mutants for HP30 and RNAi lines for both HP30 and HP30-2 were included (6)(cf. Fig. S4). Genotyping confirmed the presence of three independent alleles of homozygous *Athp30-2* plants that were designated *Athp30-2;0* (SALK_149871), *Athp30-2;1* (SALK_136524) and *Athp30-2;2* (SALK_136525)(Fig. S4A and S5). Expression studies revealed the absence of HP30-2 transcript in *Athp30-2;1* and *Athp30-2;2* seedlings, whereas *Athp30-2;0* (SALK_149871) still contained such transcript and thus was a false-positive knockout mutant not used further (Fig. S4). Light-grown *Athp30-2;1* and *Athp30-2;2* plants had no visible phenotype if grown under continuous white light illumination. However, dark-grown (etiolated) *Athp30-2;1* and *Athp30-2;2* seedlings rapidly died when exposed to white light (Fig. S4B). Agarose gel electrophoresis revealed a rapid DNA fragmentation to occur in illuminated *Athp30-2;1* and *Athp30-2;2* seedlings (Fig. S4C). *Athp30;2* (SALK_112126) mutant seedlings lacking HP30 used for comparison had no comparable phenotype and did not undergo DNA fragmentation (Fig. S4B and C). Seedling lethality was overcome by genetically complementing *Athp30-2;1* plants with the HP30-2 cDNA (Fig. S4B and C). tRNA uptake assays carried out with radio- or fluorescence-labelled $tRNA^{Leu}$, $tRNA^{Ala}$ or a mixture of cytosolic tRNAs did not reveal gross differences for *Athp30-2;1* and *Athp30-2;2*, HP30::HP30-2 RNAi as well as *Athp30;1*::*Athp30-2;2* double knock-out versus wild-type mitochondria (Figs. S6-8). *Athp30;2* and *Athp30;3* mitochondria used for comparison displayed no detectable tRNA import defects either (Figs. S6-8). Protein gel blot analyses identified some co-expression changes of HP30-2 with TIM22, TIM8, TIM9

and TIM10 to happen in *Athp30-2;1* and *Athp30-2;2* knock-out mutants that were overcome by genetically complementing these lines with the HP30-2 cDNA (Fig. S4D, panel a).

Protein sequence data

Proteins interacting with LeuRS2 were identified by sequencing and found to represent HP30-2 (encoded by At5g24650)(7), TIM22 (encoded by At1g18320 or At3g10110)(8), the mitochondrial intermembrane space import and assembly protein MIA40 (encoded by At5g23395)(9, 10), TIM8 (encoded by At5g50810), TIM9 (encoded by At3g46560) and TIM10 (encoded by At2g29530) (9,10). As shown previously (6), HP30-2, TIM22, MIA40 and TIM8-10 are part of a unique translocase in the inner membrane of Arabidopsis mitochondria involved in the import of signal sequence-less precursor proteins and maybe that of carrier proteins and β-barrel proteins.

1. Rassow J, Dekker PJ, van Wilpe S, Meijer M, Soll J (1999) The preprotein translocase of the mitochondrial inner membrane: function and evolution. *J Mol Biol* 286:105–120.
2. Rossig C *et al.* (2014) New functions of the chloroplast Preprotein and Amino acid Transporter (PRAT) family members in protein import. *Plant Signal Behavior* 9(1):e27693.
3. Kim CA, Bowie JU (2003) SAM domains: uniform structure, diversity of function. *Trends Biochem Sci* 28:625-628.
4. Aviv T *et al.* (2003) The RNA-binding SAM domain of Smaug defines a new family of post-transcriptional regulators. *Nat Struct Biol* 10:614-621.
5. Murcha MW *et al.* (2016) Plant-Specific Preprotein and Amino Acid Transporter Proteins Are Required for tRNA Import into Mitochondria. *Plant Physiol* 172(4):2471-2490.
6. Rossig C et al. (2017) HP30-2, a mitochondrial PRAT protein for import of signal sequence-less precursor proteins in Arabidopsis thaliana. *J Integr Plant Biol* 59(8):535-551.
7. Murcha MW et al. (2007) Characterization of the preprotein and amino acid transporter gene family in *Arabidopsis*. *Plant Physiol* 143, 199-212.
8. Carrie C, Murcha MW, Whelan J (2010b) An *in silico* analysis of the mitochondrial import apparatus of plants. *BMC Plant Biol* 16;10:249.
9. Carrie C et al. J (2010a) Conserved and novel functions for *Arabidopsis thaliana* MIA40 in assembly of proteins in mitochondria and peroxisomes. *J Biol Chem* 285: 36138–36148.

10. Riemer J, Fischer M, Herrmann JM (2011) Oxidation-driven protein import into mitochondria: Insights and blind spots. *Biochim Biophys Acta* 1808: 981–989.

SI Figures

```
LeuRS1  1     MsshhqilqirsdpfvlshccrhtrltssltlqsplkqpfsclpfrwrrsyrggvrssttethgskkealVSETATTS  78
LeuRS2  1     M---------------------------------------------------------------------ASES-KSY  8

LeuRS1  79    IELKRVYPFHEIEPKWQRYWEDNRIFRTP--DDVDTSKPKFYVLDMFPYPSGAgLHVGHPLGYTATDIL  145
LeuRS2  9     ARRDRLL---EIEATVRKWWEDEDVFRAEscENLPKPGEKFFSTFPFPYMNGY-LHIGHAFSLSKVDFA  73

LeuRS1  146   ARLRRMQGYNVLHPMGWDAFGLPAEQYAIE-------TG----------------------T--------------  178
LeuRS2  74    SAYHRLRGANVLLPFGFHCTGMPIKASADKLRREIEQFGnppvftaedttkvpevqeessdTialpipgqfkgkks  149

LeuRS1  179   ------------------------------HPKTTTLKNIDRFRLQLKSLGFSYDWDRELSTTE--PDYYKWTQWIFLQL  226
LeuRS2  150   kvaakaggqvyqWEIMRSFGLTDSEIANFREPSEWLYYFPPLAVEDLRAYGLGCDWRRSFVTTDVNPFFDAFVRWQMRKL  229

LeuRS1  227   YKKGLAYQAEVPVNWCPALGTVLANEEVVDGVSERG-GHPVIRKPMRQWMLKITAYADRLLEDLDELE-WPESIKEMQRN  304
LeuRS2  230   KSMGKIVKDRRYTIFSPLDGQPCADHDRATGEGVQP--------------------QEYTLIKMEVV-------------  276

LeuRS1  305   WIGRSEGAELNFSILDGEGRETdKEITVYTTRPDTLFGATYMVVAPEHQLLSYFV-------TAEQKQQVEEYKDFA  374
LeuRS2  277   --------KPFPLKLGPLEGKR-VFLAAATLRPETMYGQTNAWVLPDGKYGAYEISETeVFILTERAALNLAYQNFS  344

LeuRS1  375   SRKSDLERTELQKDKTGVFTGCYAKNPANG-DAIPIWVADYVLASYGTGAIMAVPAHDTRDN----------EFALKYNI  443
LeuRS2  345   KNPQEPSCLVELTGYD--LIGLPLRSPLSVNEIIYALPMLTILTNKGTGIVTSVPSDAPDDYMALQDLIKKPALQDKYGV  422

LeuRS1  444   PIKWVVR------NEANSSDD-AKQVYPGLGII-------------------ENSSTLETGlDINQLSSKEAALKVIEW  496
LeuRS2  423   KTEWLPTEIIPIINIPEFGDKAAEKVCLDLKIKSQNDKEKLAEAKRLTYLKGFTEGTMLIG-EFFGRKVQEIKPIIKTK  500

LeuRS1  497   AERTGNG------KKKVNYKLRDWLFA--RQRYW----GEPIPILILDESGETIAISESE-------------------  544
LeuRS2  501   LIETGEAIIYSEPEKPVMSRSGDECVV-ALTDQWYITYGESEWRKIAEECLSKMNLYSDETRHGFEHTLSWLNQWACSR  578

LeuRS1  545   ---LPLTLPELNDFTPTGTGEPPLSKAVSWVN----TVDPSTGKPAKRET----STMPQWAGSCWYYLRFMDPK  607
LeuRS2  579   SFGLGTRIPWDEQFLVESLSDSSLYMAYYTVAHIfhDGDMYKGSKSL---IRPQQMNDEVWEYLFCDGPYP---  646

LeuRS1  608   N-------PEALVDKEKEKYWSPVDVYVGGAEHAVLHLLYSRFWHKVLYDIGVVSTKEPFKCVINQGIILGevqyta  677
LeuRS2  647   -KSSDIPSAVLSEMKQEFDYWYPLDLRVSGKDLIQNHLTFFIYNH-----TALMANRNWPRGIRCNGHIML------  711

LeuRS1  678   wkdqEGNYVSADTEERLNEHQQVTIPEEKVIKSGDHFVLKEDPSIRLIpRVYKMSKSRGNVVNPDDVVLEYGADSLRLYE  757
LeuRS2  712   ----NS---------------------------------------------EKMSKSTGNFRTLRQSIEEFSATGTRFCL  742

LeuRS1  758   MFMGPFRDSKTWNTSGIEGVHRFLARTWRLVIGLPQSDGSFKDGTLVTD-DEptlEQLRTLHKCIAKVTEEIESTR  832
LeuRS2  743   ADAGDGVDDANFAFETANAAILRLTKELTWMEEVLDVESSLRTGPPSTYADK---VFENDMNIALRLTERAYKDCL  815

LeuRS1  833   FNTGISGMMEFVNAAYKWN----NQ-------PRGIIEPFVLLLSPYAPHMAEELW-SRLGHPNSLAYESFPKANP  896
LeuRS2  816   FREALKNGFYDLQAARDEYRLSCGTGGMHHDLLLKFMDVQTRLIVPICPHFADYVWrKVLNKEGCVLTAGWPPSNE  891

LeuRS1  897   D-YLKNTTIVLPVQINGKTRG-----TIEVEEGCSEDDAF-------------------------------  930
LeuRS2  892   PDLVLKSANKYLQDSIVLMRKLLQKQ--LSGSKKGAKKGAqvtavpegklkgLVYvneqfDGWRAHCLRILQ  961

LeuRS1  931   ----VLASQ----DDKLRKYL-----DGQSIKKRIY--------------------------------------------  953
LeuRS2  962   SRFDQQTCSfppdTEMLAELSatllqEGKNLKAIQKvcmpflKFKKDEAISIGtqalnlR-lpFGEIEVLQSNKDLIRRQ  1040

LeuRS1  954   --------------------------------VPGKILNVILDRtnvkvttk  973
LeuRS2  1041  LGLEEVEiysasDPddvsiagphaslltQNPPSPGSPTAIFVTStsvcpps-  1091
```

Fig. S1. Amino acid sequence alignment of pLeuRS1 and LeuRS2. Signature motives of the Rossmann fold, editing, leucine-specific domain and Zn binding site are indicated and refer to the following code.

MSP = fluorescent green
Editing domain = Bold face
Leucyl-specific (LS) domain = underlined
HIGH, DWLISR, KMSKS (catalytic site) = gray
Threonine-rich and GTG motif = Cyan
Zinc binding motifs = yellow
Boxed part of the Editing domain in Cusack et al. 2000 EMBO J 19:2351 = underline italic
The coloring scheme of the NCBI Multiple Sequence Alignment Viewer was used in the conservation method. The "cons" method uses two colors red and blue to designate highly conserved or less conserved regions in a sequence alignment.

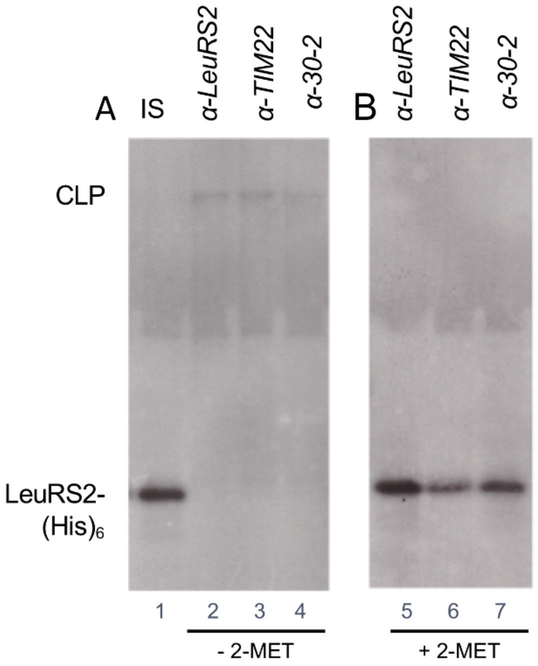

Fig. S2. Identification of HP30-2 and TIM22 in the DTNB-crosslink product of ^{35}S-LeuRS2-(His)$_6$ used as import substrate (IS). DTNB-activated ^{35}S-LeuRS2-(His)$_6$ was imported into isolated Arabidopsis mitochondria as described in Fig. 2B. Then, aliquots of the obtained crosslink product (CLP) were subjected to co-precipitation with antibodies against LeuRS2, HP30-2 or TIM22. The resulting precipitates were separated by SDS-PAGE under non-reducing conditions (- 2-MET, A) or reducing conditions (+ 2-MET, B) and ^{35}S-LeuRS2 was detected by autoradiography.

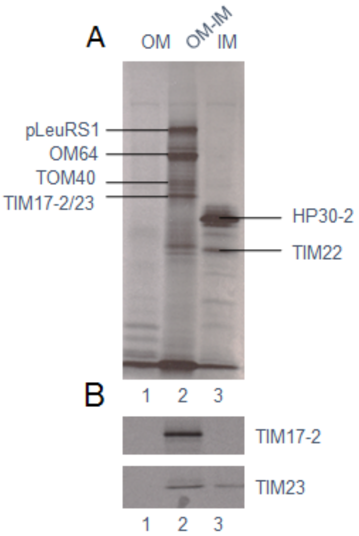

Fig. S3. Identification of proteins interacting with pLeuRS1 during its import into mitochondria. Translocation intermediates were prepared at 8°C during an in vitro-uptake reaction of pLeuRS1-$(His)_6$ into isolated Arabidopsis mitochondria. OM, OM-IM and IM fractions were isolated and separated on a 20-38% sucrose gradient and protein contained in these different fractions was detected by SDS-PAGE and protein sequencing (A) or Western blotting (B) using specific antibodies against the indicated IM marker proteins. Note the presence of TIM23 and TIM17-2 in the OM-IM fraction and the presence of HP30-2 and TIM22 in the IM fraction that is indicative for the involvement of different import machineries in pLeuRS1 versus LeuRS2 import into mitochondria (cf. Fig. 2).

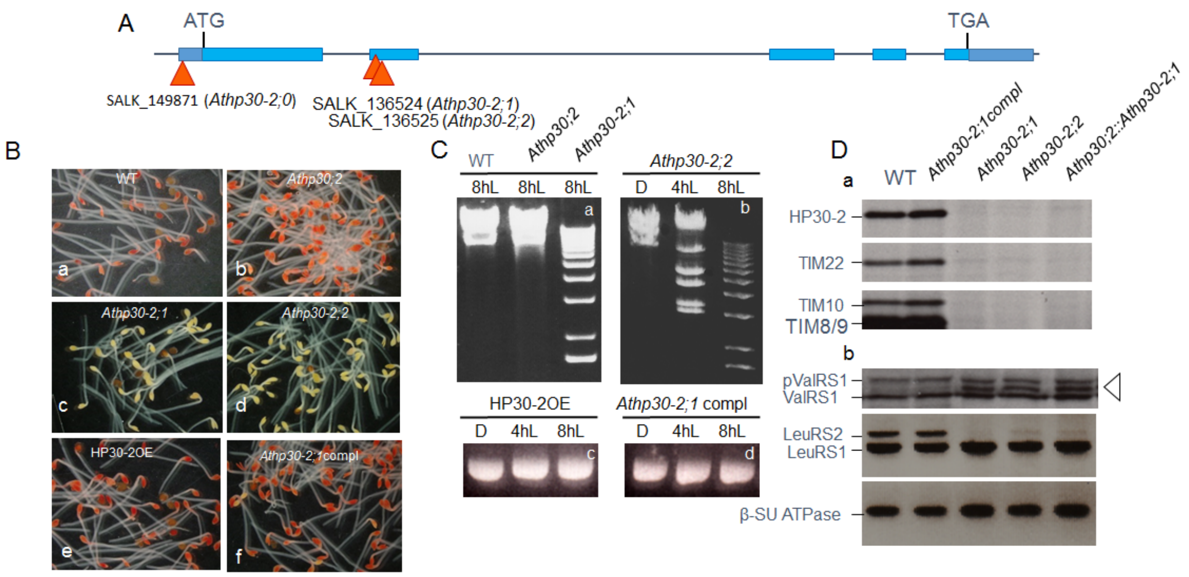

Fig. S4. Characterization of HP30-2 T-DNA insertion mutants, genetically complemented line, and wild-type plant overexpressing the *HP30-2* cDNA. A, Gene structure of At5g24650 encoding HP30-2 and positions of T-DNA insertions in *Athp30-2;0* (SALK_149871), *Athp30-2;1* (SALK_136524) and *Athp30-2;2* (SALK_136525). Exons and introns are represented by blue bars and black lines, respectively. B, Viability of 4.5 d-old dark-grown seedlings of the *Athp30-2;1* (SALK_136524, c) and *Athp30-2;2* (SALK_136525, d) mutants versus genetically complemented *Athp30-2;1* mutant (*Athp30-2;1compl*, f) line during greening. Seedling viability was assessed by tetrazolium staining. For comparison, seedling viability was tested for the *Athp30;2* (SALK_112126, b) T-DNA insertion mutant, the wild-type (WT, a) and WT seedlings overexpressing the HP30-2 cDNA (HP30-2OE, e). C, as B, but showing DNA breakage indicative of cell death in seedlings of the indicated genotypes after growth in darkness (D) and subsequent white light (L) exposures (in h). D, as B, but showing Western blot data for HP30-2, TIM22, TIM10 and TIM8/9 (a) and the amount in vitro-imported pValRS1, pLeuRS1 and LeuRS2 (b) for *Athp30-2;1* (SALK _136524), *Athp30-2;2* (SALK_136525), respective double mutants (*hp30-2;1::hp30;2*) versus genetically complemented *Athp30-2;1* (*Athp30-2;1compl*) and wild-type (WT) plants. Note that the in vitro-import reactions were carried out at 8°C and that only the incubation mixtures containing the ^{35}S-Met-labelled pLeuRS1 and LeuRS2 were treated with proteinase K, whereas those containing the ^{35}S-Met-labelled pValRS1 were not. β-SU ATPase defines the β-subunit of the mitochondrial CF_0/CF_1-ATPase used as loading

control. The arrowhead marks a partially processed form of pValRS1, migrating between the precursor and mature forms.

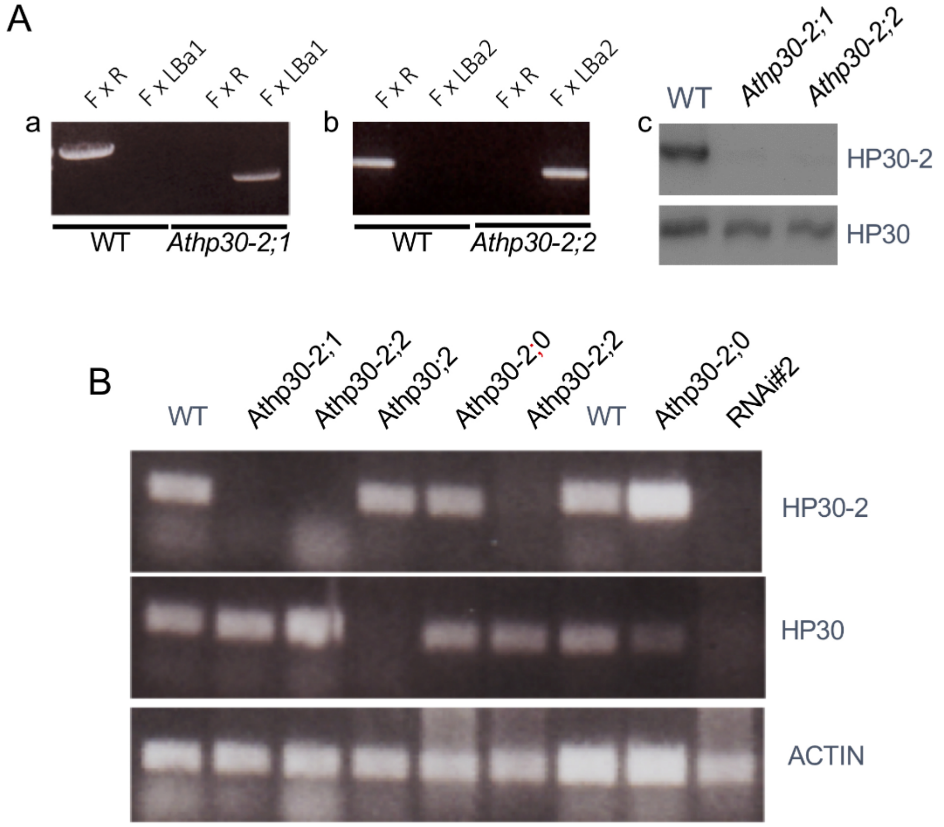

Fig. S5. Characterization of HP30 and HP30-2 knock-out mutants and RNAi lines. A, Genotyping to confirm the presence of T-DNAs in *Athp30-2;1* (SALK _136524)(panel a) and *Athp30-2;2* (SALK_136525)(panel b) and to prove the lack of HP30-2 transcript (panel c). B, Comparative RT-PCR analysis carried out for wild-type (WT) versus *Athp30;2* (SALK_112126)*, Athp30-2;0* (SALK_149871), *Athp30-2;1* (SALK_136524) and *Athp30-2;2* (SALK_136525) and *RNAi#2* plants. Note that *Athp30-2;1* (SALK_136524) and *Athp30-2;2* (SALK_136525) are true knock-out mutants, whereas *Athp30-2;0* (SALK_149871) is a false-positive knock-out mutant still containing HP30-2 transcript.

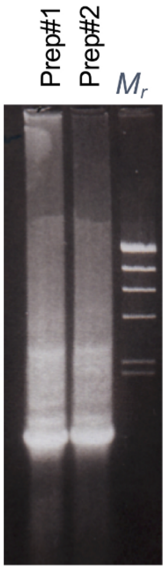

Fig. S6. Agarose gel electrophoresis of two representative tRNA preparations used in this study. The positions of molecular mass standards are indicated. RNAs were detected by ethidium bromide staining.

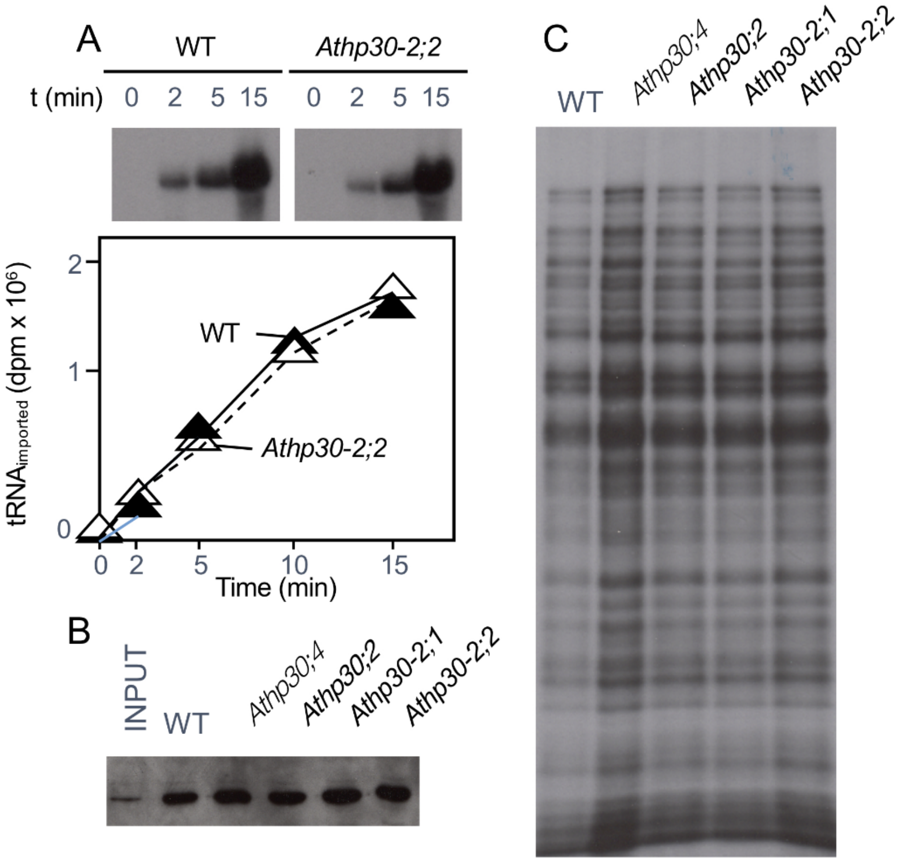

Fig. S7. tRNA uptake into isolated mitochondria of wild-type (WT), *Athp30;2* (SALK_112126), *Athp30;4* (SALK_031707), *Athp30-2;1* (SALK_136524) and *Athp30-2;2* (SALK_136525) plants. A, Time course of uptake of ^{32}P-labeled plant cytosolic tRNA^{Leu} comprising all six isoacceptors into mitochondria of wild-type (WT) and *Athp30-2;2* (SALK_136525) mitochondria. The upper rows show autoradiograms of ^{32}P-tRNA^{Leu} that had been separated on agarose gels and blotted onto nitrocellulose membranes, the lower diagram depicts respective quantitative data after scintillation counting. B, as A, but showing levels of ^{32}P-tRNA^{Ala} imported after 15 min into mitochondria of the indicated mutants versus wild-type (WT). 'Input' defines a 10% input standard. C, Patterns of ^{35}S-labeled mitochondrial proteins synthesized in organello.

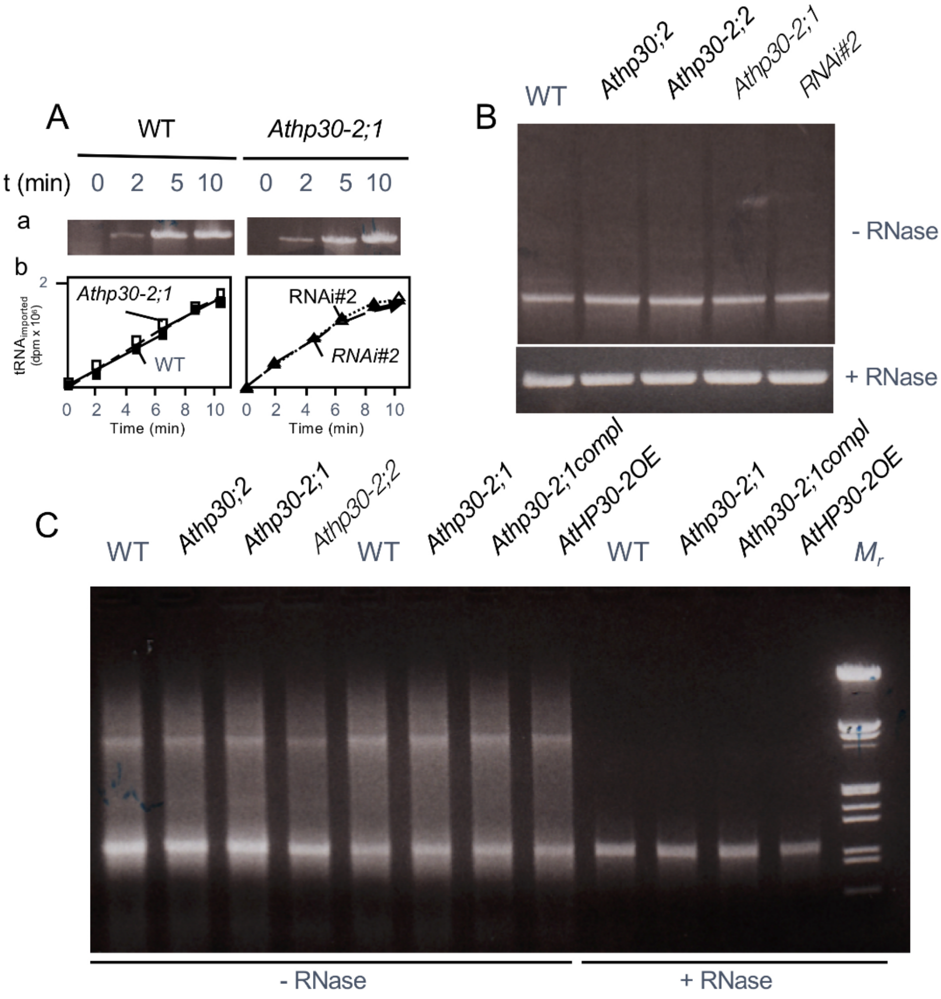

Fig. S8. tRNA uptake into isolated mitochondria. A, Time course of uptake of total cytosolic tRNA into mitochondria from wild-type (WT) versus *Athp30-2;1* (SALK_136524) seedlings, using either fluorescence-labeled tRNAs (panel a) or radioactively-labeled tRNAs (panel b). B, as A, but depicting the amounts of fluorescence-labeled tRNA incorporated into isolated mitochondria of wild-type (WT), *Athp30;2* (SALK_112126), *Athp30-2;1* (SALK_136524), *Athp30-2;2* (SALK_136525) and *RNAi#2* seedlings. Upper and lower raws show the amounts of tRNA incorporated into mitochondria before and after RNase treatment. C, as A, but depicting the amounts of fluorescence-labeled tRNA incorporated into isolated mitochondria of wild-type (WT), *Athp30;2* (SALK_112126), *Athp30-2;1* (SALK_136524) and *Athp30-2;2* (SALK_136525), *Athp30-2;1* (SALK_136524) plants re-expressing HP30-2 (*Athp30-2;1compl*), and wild-type (WT) plants overexpressing HP30-2 (AtHP30-2OE). Samples were treated with or without RNase, as indicated. Positions of molecular mass standards are highlighted.

```
NP_172913.1     -------MS------L--LFLRRAKPLFVSCCSATHSRSSFLSPTLTNQLVRSFHGSRT-   44
AAA35207.1      MNKWLNTLSKTFTFRLLNCHYRRSLPL----CQNFS-----LKKSLTHNQVRFFKMSDLD   51
                       :*      *    . **: **    *.         *. :**:: ** *: *

NP_172913.1     ----MSESEKKILTEEELERKKKKEEKAKEKELKKQKALEKERLAEKLAKQAKDGTNVPK   100
AAA35207.1      NLPPVDPKTGEVIINPLKEDGSPKT----PKEIEKEK-KKAEKLLKFAAKQAKKNAAATT   106
                    :. .  ::: :   *  . *      **::*:*  : *:* :: *****..: . .

NP_172913.1     KSAKKSSKRDASEENPEDFVDPETPLGERK---RLSSQMAKQYSPATVEKSWYAWWEKSD   157
AAA35207.1      GASQKKPKKKKEVEPIPEFIDKTVP-GEKKILVSLDDPALKGYNPANVESSWYDWWIKTG   165
                 ::*. *:. . *    :*:*  .* **:*     *..   * *.**.**.*** ** *:.

NP_172913.1     LFKADAKS-----SKPPFVIVLPPPNVTGALHIGHALTSAIEDTIIRWKRMSGYNALWVP   212
AAA35207.1      VFEPEFTADGKVKPEGVFCIPAPPPNVTGALHIGHALTIAIQDSLIRYNRMKGKTVLFLP   225
                :*: : .:      :  * *  **************** **:*::**::**.* ..*::*

NP_172913.1     GVDHAGIATQVVVEKKIMRDRGMTRHDVGREEFVKEVWKWKNQYGGTILTQLRRLGASLD   272
AAA35207.1      GFDHAGIATQSVVEKQIWAKDRKTRHDYGREAFVGKVWEWKEEYHSRIKNQIQKLGASYD   285
                *.******** ****:*  .   **** *** ** :**:**::* . * .*:::**** *

NP_172913.1     WSRECFTMDEQRSKAVTEAFVRLYKEGLIYRDIRLVNWDCILRTAISDVEVEYIDIKEKT   332
AAA35207.1      WSREAFTLSPELTKSVEEAFVRLHDEGVIYRASRLVNWSVKLNTAISNLEVENKDVKSRT   345
                ****.**:. : :*:* ******:.**:***  *****.  *.****:***  *:*.:*

NP_172913.1     LLKVPGYEKPVEFGLLTSFAYPLEGGLGEVIVATTRVETMLGDTAIAIHPDDARYKHLHG   392
AAA35207.1      LLSVPGYDEKVEFGVLTSFAYPVIGSDEKLIIATTRPETIFGDTAVAVHPDDDRYKHLHG   405
                **.****:: ****:*******: *.  ::*:**** **::****:*:**** *******

NP_172913.1     KFAVHPFNGRKLPIICDGILVDPNFGTGCVKITPAHDPNDCEVGKRHKLEFINIFTDDGK   452
AAA35207.1      KFIQHPFLPRKIPIITDKEAVDMEFGTGAVKITPAHDQNDYNTGKRHNLEFINILTDDGL   465
                **  ***  **:*** *   ** :****.******** ** :.****:******:****

NP_172913.1     INTNGGSDFAGMPRFAAREAVVEALQKQGLYRGAKNNEMRLGLCSRTNDVIEPMIKPQWY   512
AAA35207.1      LNEECGPEWQGMKRFDARKKVIEQLKEKNLYVGQEDNEMTIPTCSRSGDIIEPLLKPQWW   525
                :* : * :: ** ** **: *:* *::.** * ::*** :  ***:.*:***::****:

NP_172913.1     VNCSMIGKEALDVAITDENKKLEFVPKQYTAEWRRWLENIRDWCISRQLWWGHRIPAWYA   572
AAA35207.1      VSQSEMAKDAIKVVK---DGQITITPKSSEAEYFHWLGNIQDWCISRQLWWGHRCPVYFI   582
                *. * :.*:*:.*.     : :: :.**.  **: :** **:************ *.::

NP_172913.1     TLEEDQLKEVGAYSDHWVVARTEDDAREEAAQKFLGKKFELTRDPDVLDTWFSSGLFPLS   632
AAA35207.1      NIEGEEHDRI--DGDYWVAGRSMEEAEKKAAAKYPNSKFTLEQDEDVLDTWFSSGLWPFS   640
                .:* :: ..:   .*:**..*: ::*.::** *: ..** * :* ***********:*:*

NP_172913.1     VLGWPDVTDDFKAFYPTSVLETGHDILFFWVARMVMMGMKLGGEVPFSKVYFHPMIRDAH   692
AAA35207.1      TLGWPEKTKDMETFYPFSMLETGWDILFFWVTRMILLGLKLTGSVPFKEVFCHSLVRDAQ   700
                .****: *.*::*** *:**** *******:**::*:** *.***.:*: * ::***:

NP_172913.1     GRKMSKSLGNVIDPLEVINGVTLEGLHKRLEEGNLDPKEVIVAKEGQVKDFPNGIPECGT   752
AAA35207.1      GRKMSKSLGNVIDPLDVITGIKLDDLHAKLLQGNLDPREVEKAKIGQKESYPNGIPQCGT   760
                ***************:**.*:.*:.** :* :******:**  ** ** :.:*****:***

NP_172913.1     DALRFALVSYTAQSDKINLDILRVVGYRQWCNKLWNAVRFAMMKLGDGYTPPQTLSPE-T   811
AAA35207.1      DAMRFALCAYTTGGRDINLDILRVEGYRKFCNKIYQATKFALMRLGDDYQPPATEGLSGN   820
                **:**** :**: . .******** ***::***::*.:**:*:***.* ** * . . .

NP_172913.1     MPFSCQWILSVLNKAISKTVVSLDAFEFSDAANTIYAWWQYQFCDVYIEAIKPYFAGDNP   871
AAA35207.1      ESLVEKWILHKLTETSKIVNEALDKRDFLTSTSSIYEFW-YLICDVYIENSKYLIQEG--   877
                  :  :***  *.:: . .  :**  :*  ::.:** :* * :******  *  :  .

NP_172913.1     TFASERAHAQHALWISLETGLRLLHPFMPFVTEELWQRLPAPKDTERKASIMICDYPSAI   931
AAA35207.1      -SAIEKKSAKDTLYILLDNALKLIHPFMPFISEEMWQRLPKRS-TEKAASIVKASYPVYV   935
                  * *:  *:.:*:* *:..*:*:******::**:*****  . **: ***: ..**  :

NP_172913.1     ENWSNEKVESEMDTVLATVKCMRALRAGLLEKQKNERLPAFALCENNVTSEIVKSHE---   988
AAA35207.1      SEYDDVKSANAYDLVLNITKEARSLLSEYNIL-KNGK--VF--VESNH-EEYFKTAEDQK   989
                .::.: *  .  * **  .*  *:* :       ** :  .*   *.*  .* .*: *

NP_172913.1     LEIRTLANLSSLEVVSKGQHAAPPGSSVETVNENLKVYLEVDGAINTEAEQEKIRNKIGE   1048
AAA35207.1      DSIVSLIKAIDEVTVVRDASEIPEGCVLQSVNPEVNVHLLVKGHVDIDAEIAKVQKKLEK   1049
                 .* :* :  .  .* :.     * *. ::**  ::*:* *.* :: :**  *:::*: :

NP_172913.1     LQKQKEKLQKMMSVSTYEEKVPANIKEDNANKLAKILQEFDFFEKESARLAAETSNSGNQ   1108
AAA35207.1      AKKSKNGIEQTINSKDYETKANTQAKEANKSKLDNTVAEIEGLEATIENLKRLKL-----   1104
                 :*.*: ::: :. . ** *. :: ** * .** : : *:: :*    .*    .
```

Note: Asterisk (*) = fully conserved residue; Colon (:) = groups of strongly similar properties; Period (.) = groups of weakly similar properties. NP_172913.1 (At1g14610 or OAP18194.1) = Arabidopsis ValRS; AAA35207 = Yeast ValRS. Underline = TRBD

Residue	Colour	Property
AVFPMILW	RED	Small (small+ hydrophobic (incl.aromatic -Y))
DE	BLUE	Acidic
RK	MAGENTA	Basic - H
STYHCNGQ	GREEN	Hydroxyl + sulfhydryl + amine + G
Others	Grey	Unusual amino/imino acids etc

Fig. S9. Amino acid sequence alignment of yeast pValRS1 (AAA35207) with the closest relative from *Arabidopsis thaliana* (encoded by At1g14610). Sequence comparison was performed

using CLUSTAL O(1.2.4) multiple sequence alignment at https://www.ebi.ac.uk/Tools/msa/clustalo/.

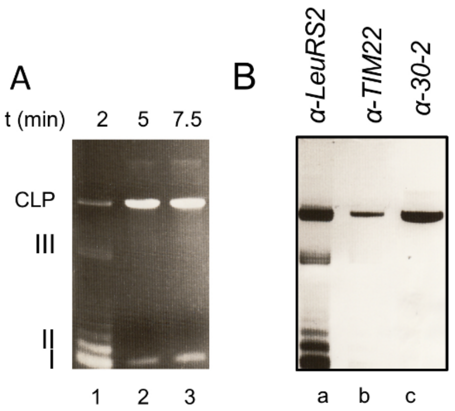

Fig. S10. tRNA binding to the HP30-2- and TIM22-containing DTNB-crosslink product of ^{35}S-LeuRS2-(His)$_6$. A, DTNB-activated ^{35}S-LeuRS2-(His)$_6$ was imported into isolated Arabidopsis mitochondria as described in Fig. 2B in assay mixtures containing tRNA^{Leu}. Then, the obtained crosslink products (designated I-III versus CLP) were detected upon agarose gel electrophoresis and fluorescence excitation at 260 nm. B, Aliquots of assay mixture obtained at 7.5 min were co-precipitated with antibodies against LeuRS2, HP30-2 or TIM22. The resulting precipitates were separated as before and respective protein gel blots probed with antibodies against LeuRS2 using an enhanced chemiluminescence (ECL) detection kit. Note the presence of tRNA in the main CLPs in A.

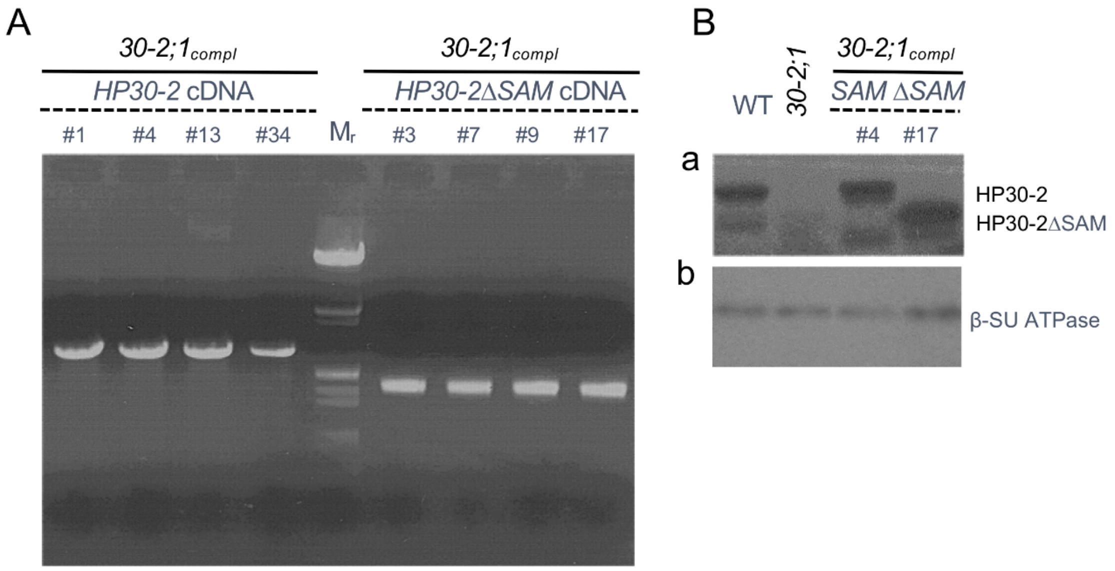

Fig. S11. Characterization of transgenic *Athp30-2;1* lines expressing the HP30-2 and HP30-2ΔSAM cDNAs.

A, Reverse transcription-polymerase chain reaction (RT-PCR) analysis of HP30-2 transcript (left) and HP30-2ΔSAM transcript (right) in the different genetically complemented lines (numbered). M_r defines molecular mass standards. B, panel a, Western blot analysis to confirm the stable expression of the HP30-2 and HP30-2ΔSAM proteins in the selected transgenic *Athp30-2;1::HP30-2SAM* (lines #4) and *Athp30-2;1::HP30-2ΔSAM* (line #17), respectively. For comparison, mitochondrial protein extracts from wild-type plants (WT) and parental *Athp30-2;1* mutant plants were included. Western blotting of SDS-PAGE-resolved proteins was carried out with HP30-2 antiserum [1]. Panel b shows a replicate gel blot analysis that was probed with an antiserum against the β-subunit of the mitochondrial CF_0/CF_1-ATPase.

[1] Rossig C, Gray J, Valdes O, Rustgi S, von Wettstein D, Reinbothe C, Reinbothe S (2017) HP30-2, a mitochondrial PRAT protein for import of signal sequence-less precursor proteins in *Arabidopsis thaliana*. J Integr Plant Biol 59(8):535-551.

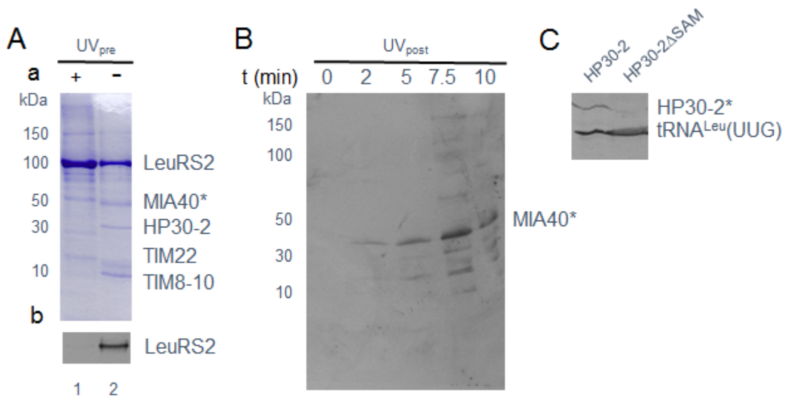

Fig. S12. Effect of $tRNA^{Leu}$(UUG) crosslinking on the mitochondrial import of LeuRS2. LeuRS2-$(His)_6$ was incubated in darkness with $tRNA^{Leu}$(UUG) to allow the formation of protein-tRNA complexes. For subsequent SDS-PAGE and autoradiography, ^{32}P-labeled $tRNA^{Leu}$(UUG) was used, whereas samples for protein sequencing contained non-radioactive $tRNA^{Leu}$(UUG). The established tRNA-protein complexes then were purified by Ni-NTA affinity chromatography. Either before (A) or during (B) the following mitochondrial import reaction, crosslinking of tRNA to LeuRS2 was induced by UV light exposure. Controls were kept in darkness. A, panel a, Pattern of mitochondrial outer and inner membrane proteins co-purifying with LeuRS2-$(His)_6$ at 8°C in assays containing the photocrosslinked tRNA (+ UV_{pre}) and non-photocrosslinked tRNA (- UV_{pre}). Protein detection was made by SDS-PAGE and Commassie-staining. Protein identification was made by protein sequencing. The band designated MIA40* contained both MIA40 and HP30-2 peptides after sequencing and thus was presumably formed by virtue of MIA40's intrinsic protein disulfide isomerase activity linking it to some of HP30-2's intrinsic Cy residues. Panel b, as panel a, but showing the amount of LeuRS2-$(His)_6$ obtained after an import reaction at 23°C. Protein detection was made by SDS-PAGE and Western blotting using (His)-tag antibodies. B, Photocrosslinking of mitochondrial outer and inner membrane proteins including HP30-2 during an import reaction at 23°C of the tRNA-complexed LeuRS2-$(His)_6$ into mitochondria. Photocrosslinking was induced at the indicated time points of the LeuRS2-$(His)_6$ import reaction. Protein detection was made by SDS-PAGE and autoradiography. C, Binding of ^{32}P-$tRNA^{Leu}$(UUG) to wheat germ-expressed and purified HP30-2 and HP30-2ΔSAM proteins. Binding assays were carried out as described in A, but without a subsequent step of affinity chromatography. Free and LeuRS2-bound ^{32}P-$tRNA^{Leu}$(UUG) was detected by PAA/urea mini gel electrophoresis and autoradiography. COMPARE with previous data.

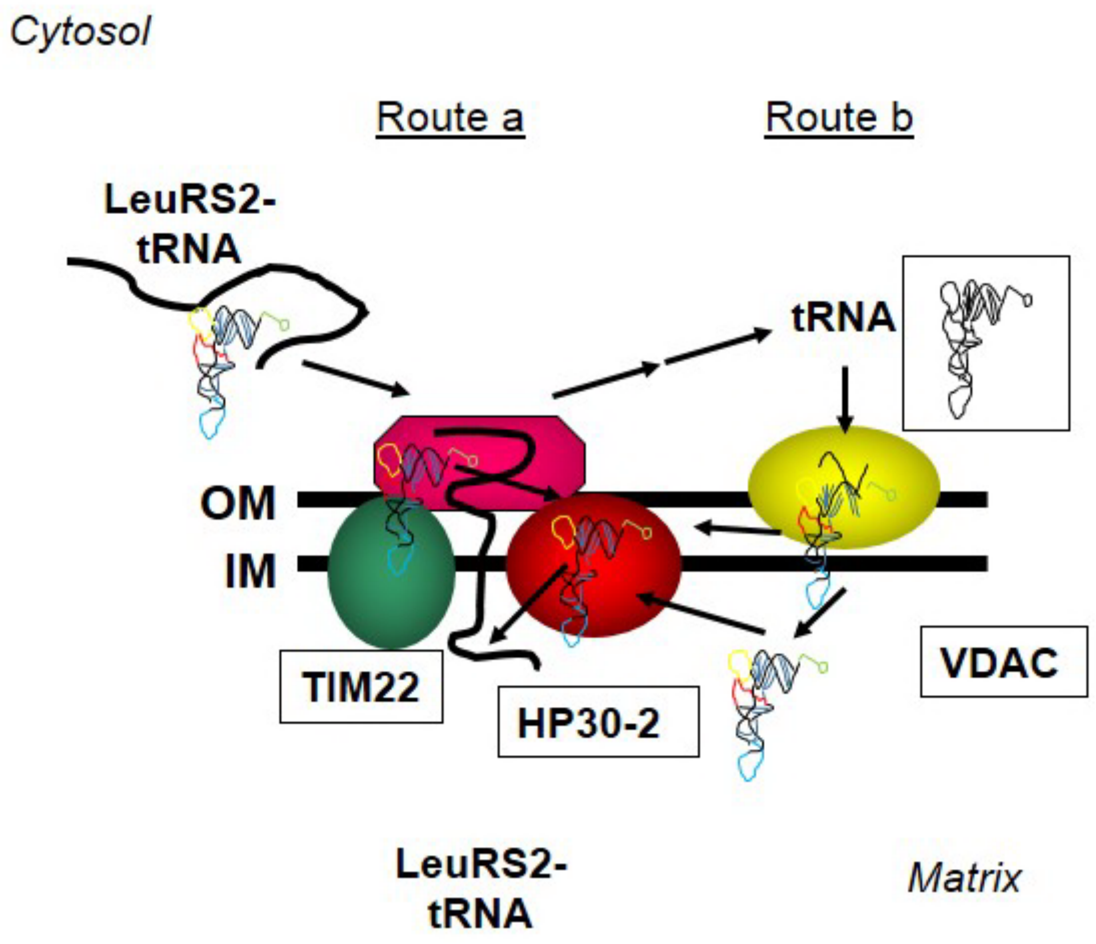

Fig. S13. Model on the co-import of LeuRS with $tRNA^{Leu}$.

In route a, LeuRS2 is supposed to bind $tRNA^{Leu}$ in the cytosol before interacting with mitochondria in the import reaction. Upon translocation, $tRNA^{Leu}$ is presumed to be stripped from the unfolded LeuRS2 and to directly bind to HP30-2. In the alternative route b, the stripped $tRNA^{Leu}$ would first pass the outer mitochondrial membrane through VDAC before being bound to HP30-2. In both models, HP30-2 is thought to function as tRNA relay in the inner mitochondrial membrane that provides LeuRS loaded with $tRNA^{Leu}$. Abbreviations: OM, outer mitochondrial membrane; IM, outer mitochondrial membrane; VDAC, voltage-dependent anion channel.

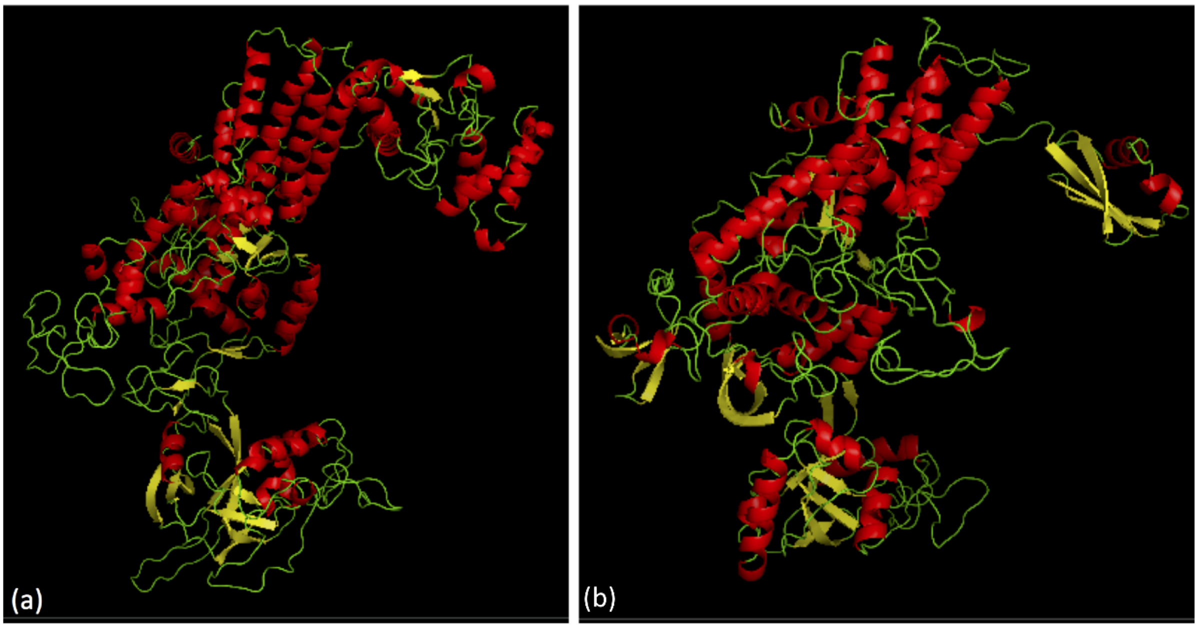

Fig. S14. The 3D-models of (a) LeuRS2 and (b) LeuRS1 were computed by the SWISS-MODEL server homology modelling pipeline. Notice the subtle differences in the editing domains of the two enzymes.

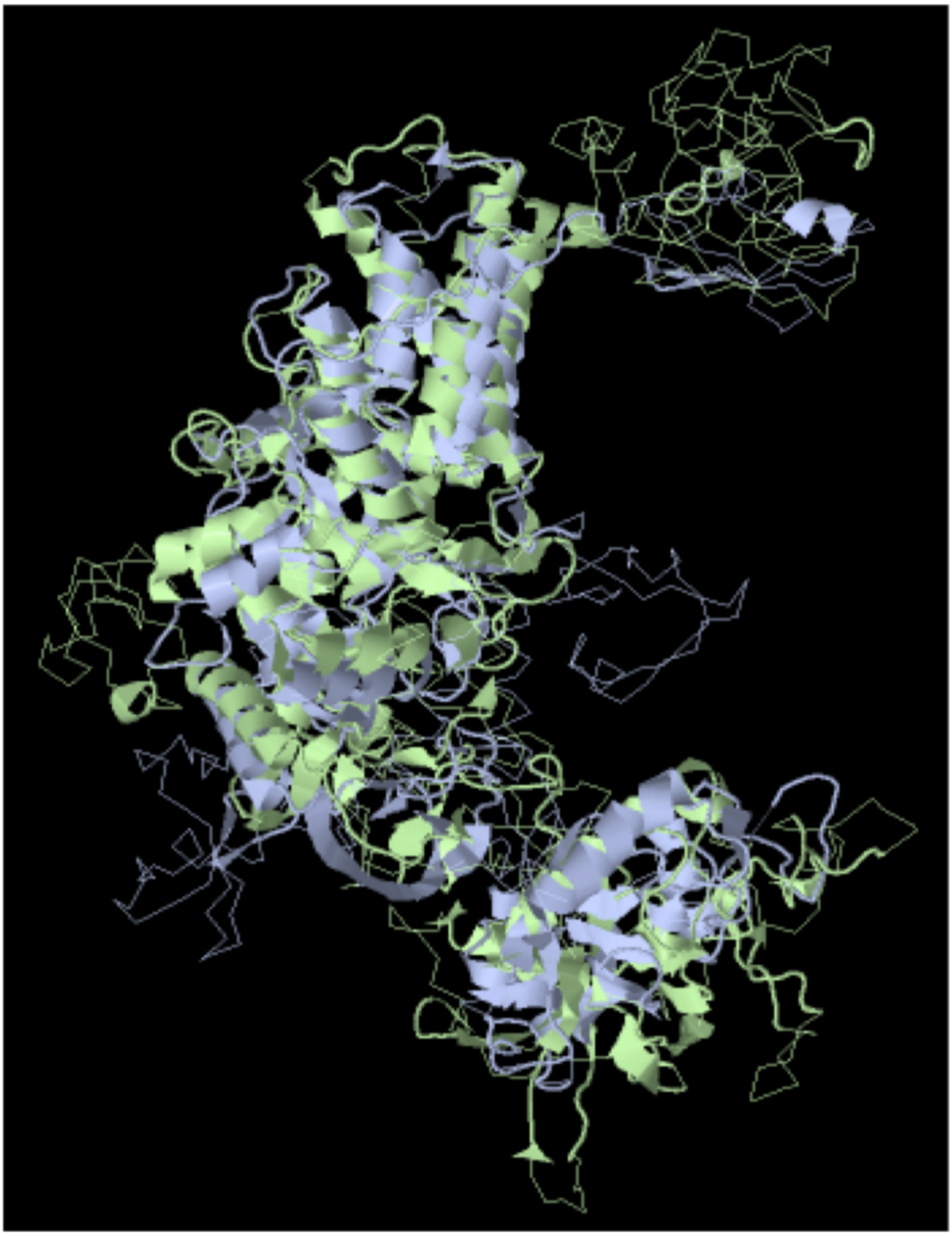

Fig. S15. The predicted 3D-models of LeuRS1 (blue) and LeuRS2 (green) were overlaid on each other using RaptorX. It highlighted the subtle differences in the structures of the two otherwise closely related enzymes.

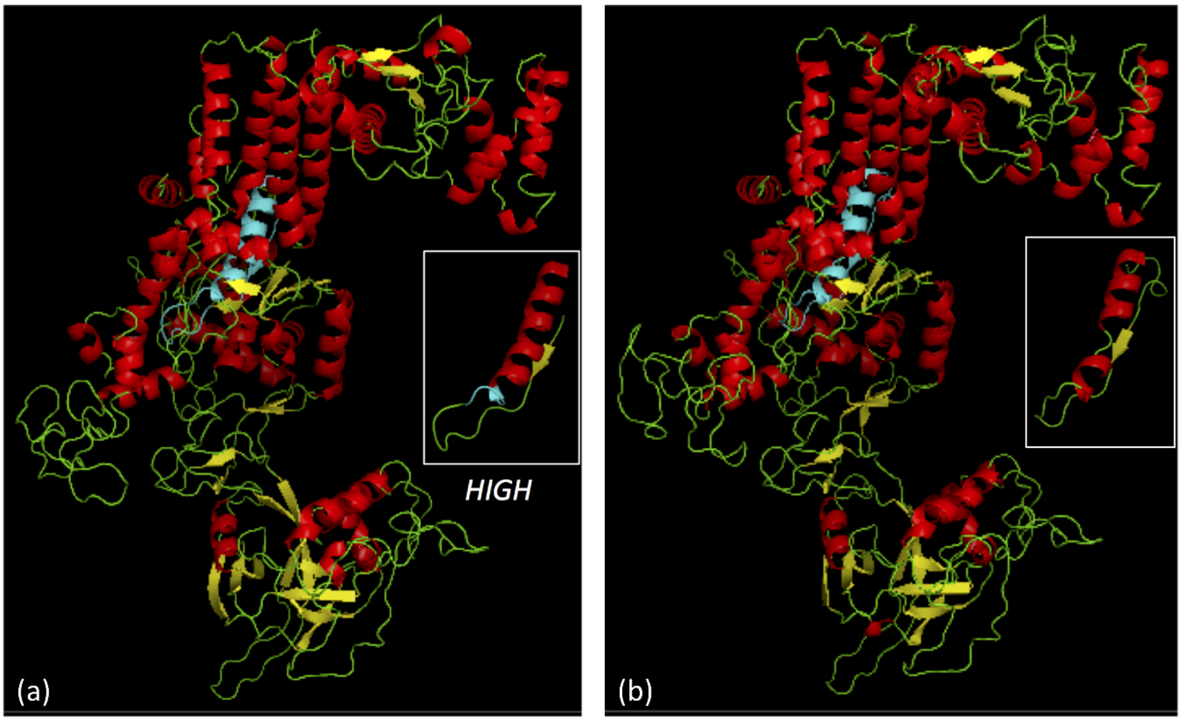

Fig. S16. 3D-model of LeuRS2 with (a) and without (b) the HIGH-motif. β-strands are shown in yellow, α-helices in red and the connecting loops in green. Cyan = part of protein carrying the selected domain, which is projected in the inset. In inset, cyan = the selected domain

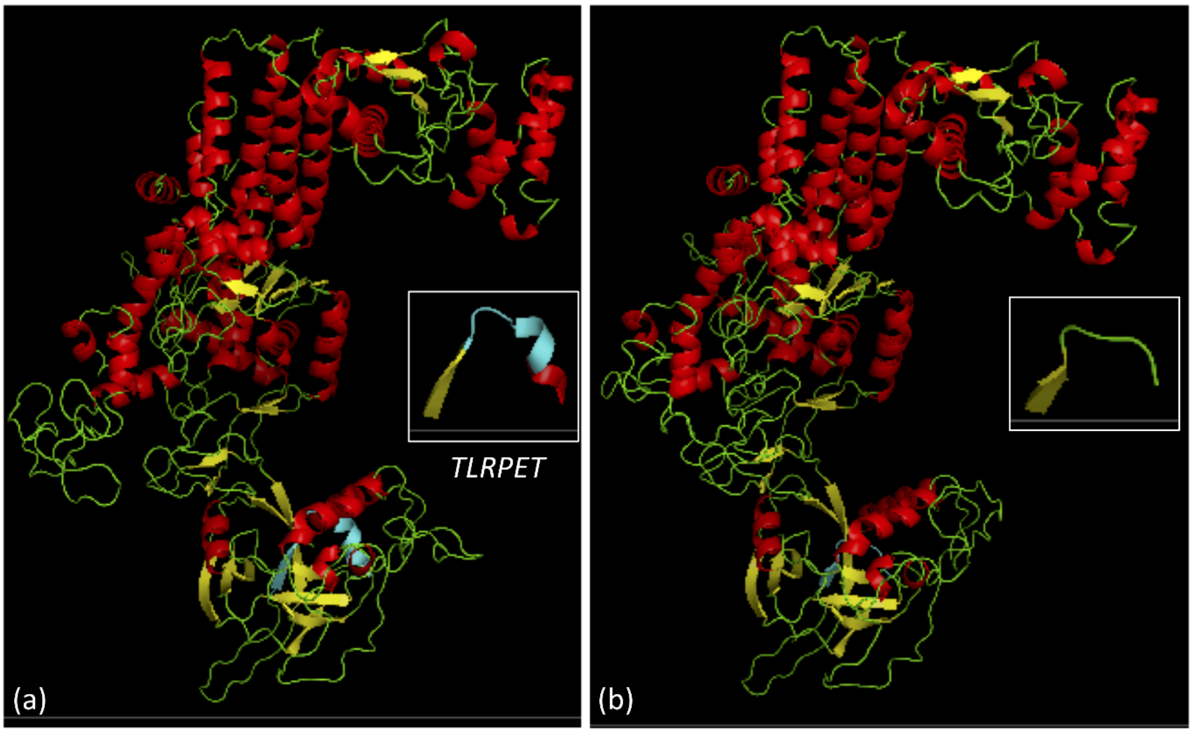

Fig. S17. 3D-model of LeuRS2 with (a) and without (b) the TLRPET-motif. β-strands are shown in yellow, α-helices in red and the connecting loops in green. Cyan = part of protein carrying the selected domain, which is projected in the inset. In inset, cyan = the selected domain

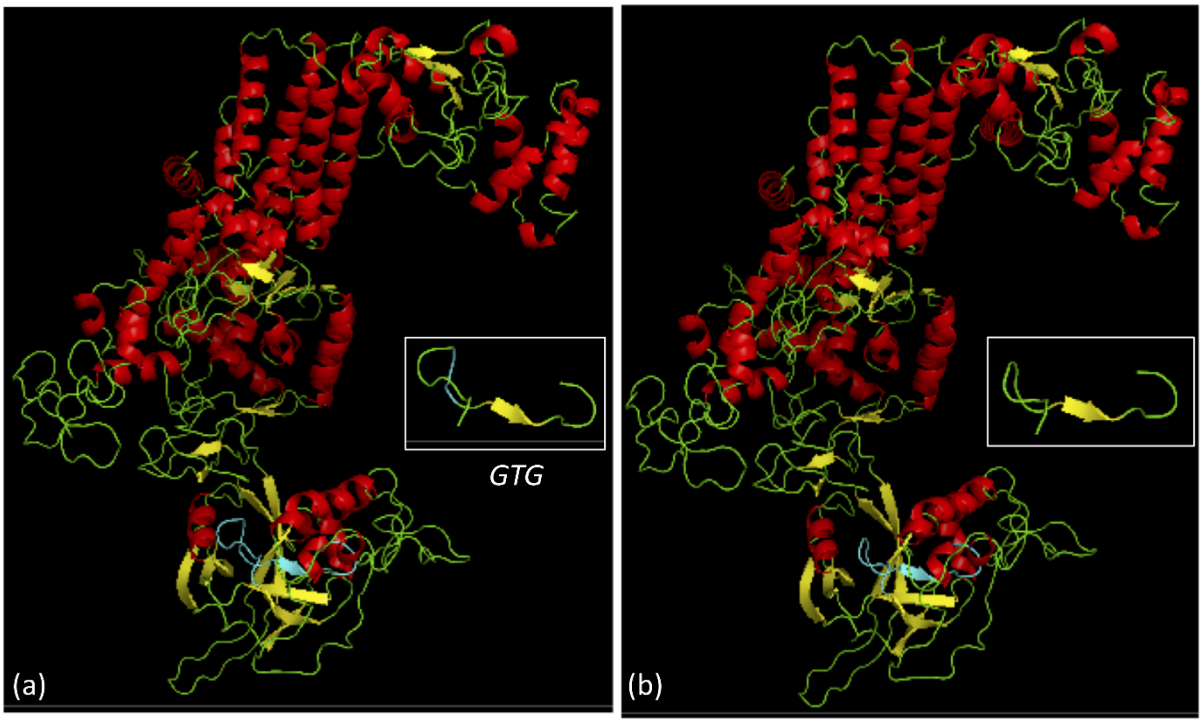

Fig. S18. 3D-model of LeuRS2 with (a) and without (b) the GTG-motif. β-strands are shown in yellow, α-helices in red and the connecting loops in green. Cyan = part of protein carrying the selected domain, which is projected in the inset. In inset, cyan = the selected domain

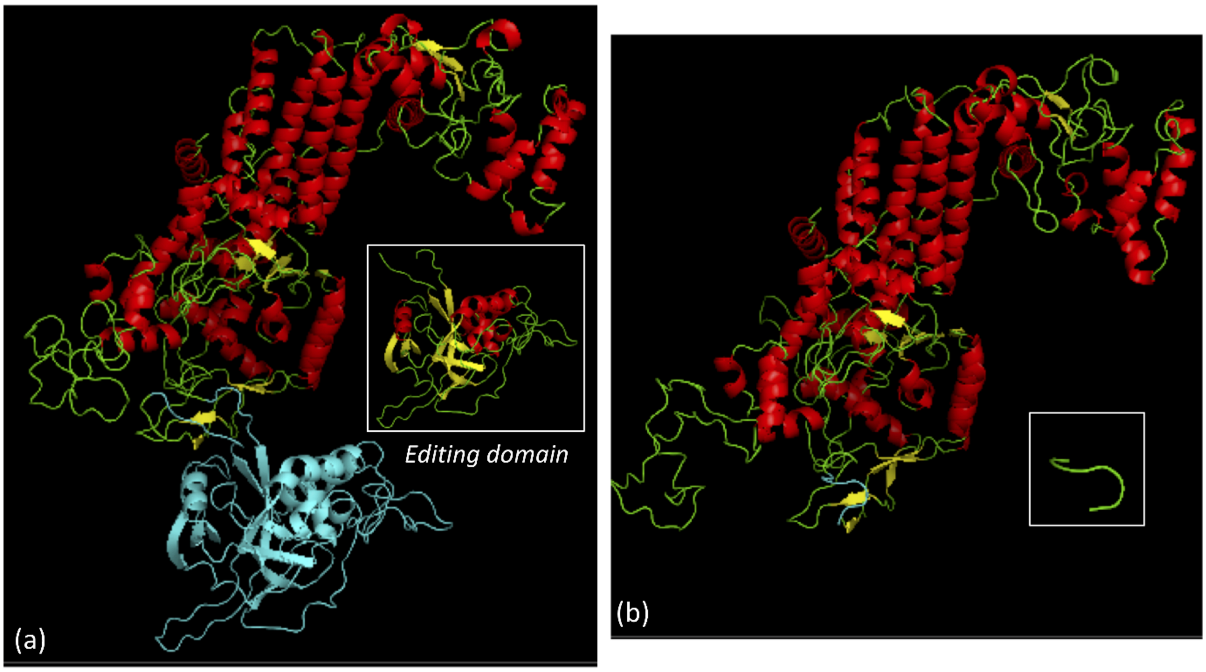

Fig. S19. 3D-model of LeuRS2 with (a) and without (b) the editing domain. β-strands are shown in yellow, α-helices in red and the connecting loops in green. Cyan = part of protein carrying the selected domain, which is projected in the inset. In inset, cyan = the selected domain

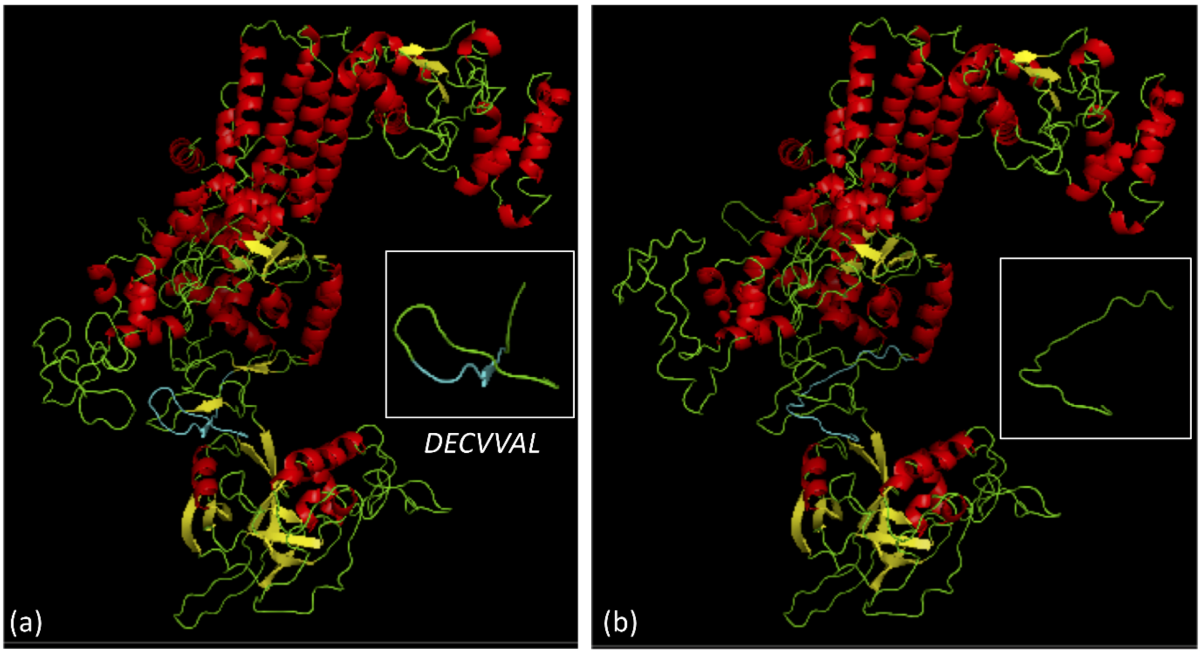

Fig. S20. 3D-model of LeuRS2 with (a) and without (b) the DECVVAL-motif. β-strands are shown in yellow, α-helices in red and the connecting loops in green. Cyan = part of protein carrying the selected domain, which is projected in the inset. In inset, cyan = the selected domain

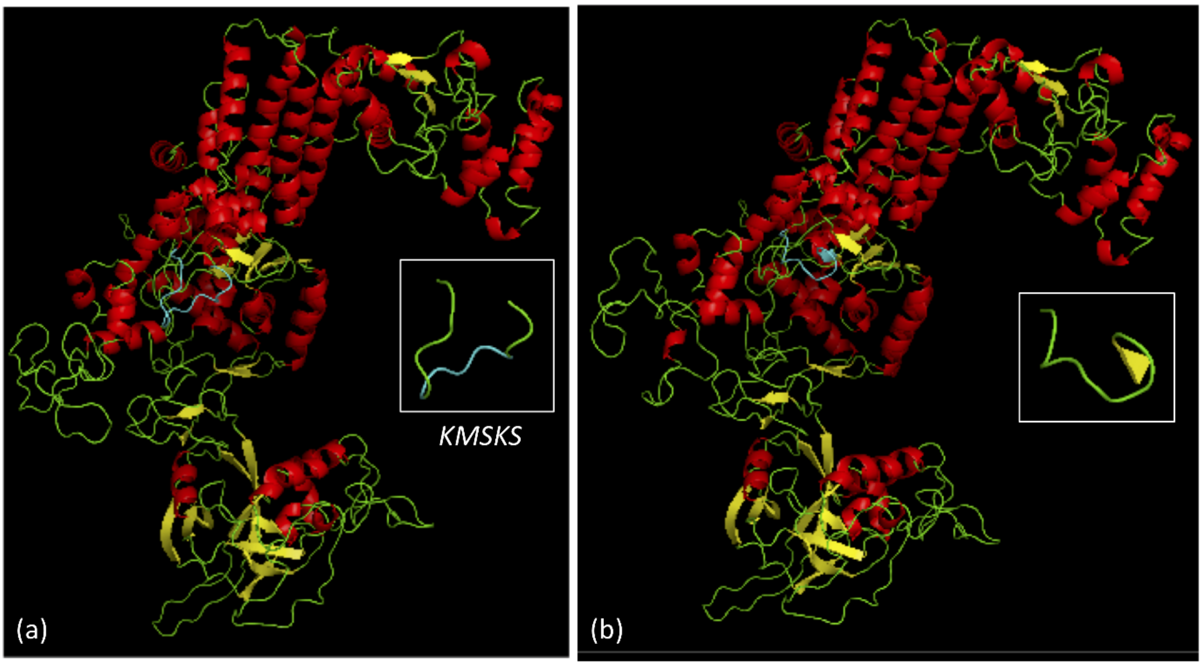

Fig. S21. 3D-model of LeuRS2 with (a) and without (b) the KMSKS-motif. β-strands are shown in yellow, α-helices in red and the connecting loops in green. Cyan = part of protein carrying the selected domain, which is projected in the inset. In inset, cyan = the selected domain

Table S1 Binding of $tRNA^{Leu}$ isoacceptors to and stimulation of LeuRS2 import into mitochondria

	$tRNA^{Leu}$					
	UUA	UUG	CUU	CUC	CUA	CUG
tRNA binding						
LeuRS2 (n-fold)	9.80	10.20	2.95	2.90	4.95	5.05
LeuRS1 (n-fold)	1.04	1.02	1.05	1.01	0.96	0.94
Import						
LeuRS2 (n-fold)	9.50	10.60	3.03	2.95	4.94	5.12
LeuRS1 (n-fold)	1.02	0.98	1.01	1.06	0.98	0.88

tRNA binding to and stimulation of mitochondrial LeuRS2 import refer to assays run in the absence of added tRNA, set as 1.00 (100%).

Table S2 Lack of stimulation of LeuRS2 import by yeast $tRNA^{Ser}$ and bacterial $tRNA^{Tyr}$ isoacceptors

	$tRNA^{Ser1)}$				$tRNA^{Tyr}$	
	UCU	UCC	UCA	AGU	UAU	UAC
LeuRS2 (n-fold)	1.02	0.98	1.02	0.97	1.02	1.04
LeuRS1 (n-fold)	0.98	1.04	0.97	0.98	1.01	1.03

Mitochondrial import data refer to assays run in the absence of added tRNA, set as 1 (100%).

1) The two other isoacceptors, $tRNA^{Ser}$-UCG and $tRNA^{Ser}$-AGC, were not included.

Table S3 Carrier-free import of plant $tRNA^{Leu}$, yeast $tRNA^{Ser}$ and bacterial $tRNA^{Tyr}$ isoacceptors into isolated Arabidopsis mitochondria

	$tRNA^{Leu}$		$tRNA^{Ser}$		$tRNA^{Tyr}$	
	UUA	CUA	UCA	AGU	UAU	UAC
hp30-2;1						
(n-fold)	1.03	0.98	0.96	1.02	1.02	0.99
hp30-2;2						
(n-fold)	0.99	1.02	0.97	1.01	1.03	1.02

Mitochondrial tRNA uptake data refer to those determined for the wild-type control, set as 1 (100%).

Table S4 Mutation of LeuRS2 and effect on its tRNA binding and mitochondrial import capabilities

		$tRNA^{Leu}$ binding	Import
<u>LeuRS2</u>			
Rossmann-fold deletions			
1	HIGH	88	92
2	DWLISR	78	76
3	KMSKS	68	72
4	HIGH+DWLISR	56	64
5	HIGH+KMSKS	62	58
6	DWLISR+KMSKS	44	38
7	HIGH+DWLISR+KMSKS	14	12
tRNA editing domain deletions			
8	Threonine-rich domain	86	88
9	GTG	84	82
10	Threonine-rich domain and GTG	66	64

tRNA binding and mitochondrial import data refer to those determined for the wild-type protein, set as 100%.
